# Terahertz metamaterials and systems based on rolled-up 3D elements: designs, technological approaches, and properties

**DOI:** 10.1038/srep43334

**Published:** 2017-03-03

**Authors:** Victor Ya. Prinz, Elena V. Naumova, Sergey V. Golod, Vladimir A. Seleznev, Andrey A. Bocharov, Vitaliy V. Kubarev

**Affiliations:** 1 Rzhanov Institute of Semiconductor Physics, Russian Academy of Science, Siberian Branch, Novosibirsk, 630090, Russia; 2 Budker Institute of Nuclear Physics, Russian Academy of Science, Siberian Branch, Novosibirsk, 630090, Russia

## Abstract

Electromagnetic metamaterials opened the way to extraordinary manipulation of radiation. Terahertz (THz) and optical metamaterials are usually fabricated by traditional planar-patterning approaches, while the majority of practical applications require metamaterials with 3D resonators. Making arrays of precise 3D micro- and nanoresonators is still a challenging problem. Here we present a versatile set of approaches to fabrication of metamaterials with 3D resonators rolled-up from strained films, demonstrate novel THz metamaterials/systems, and show giant polarization rotation by several chiral metamaterials/systems. The polarization spectra of chiral metamaterials on semiconductor substrates exhibit ultrasharp quasiperiodic peaks. Application of 3D printing allowed assembling more complex systems, including the bianisotropic system with optimal microhelices, which showed an extreme polarization azimuth rotation of 85° with drop by 150° at a frequency shift of 0.4%. We refer the quasiperiodic peaks in the polarization spectra of metamaterial systems to the interplay of different resonances, including peculiar chiral waveguide resonance. Formed metamaterials cannot be made by any other presently available technology. All steps of presented fabrication approaches are parallel, IC-compatible and allow mass fabrication with scaling of rolled-up resonators up to visible frequencies. We anticipate that the rolled-up meta-atoms will be ideal building blocks for future generations of commercial metamaterials, devices and systems on their basis.

Metamaterials extended the available range of electromagnetic properties^1^,^2^,^3^,^4^,^5^,^6^ far beyond the limits of ever known materials. This extraordinary freedom in tailoring of electromagnetic properties is the most intriguing point in the concept of metamaterials that makes a ground for unique possibilities in manipulation of electromagnetic radiation and opens the way to unique potential applications from subwavelength focusing to invisibility cloaking^7^,^8^,^9^,^10^,^11^,^12^. There has been a plenty of publications devoted to metamaterials and metasurfaces, i.e. monolayer metamaterials (see reviews^6^,^13^,^14^), but very few of real-world applications^8^,^15^,^16^,^17^,^18^,^19^,^20^,^21^,^22^. Metamaterials are at the stage of active development^6^,^10^,^23^. The development scheme of THz and optical metamaterials can be outlined as the progress from metamaterials with planar resonators^8^,^14^,^24^,^25^,^26^,^27^, to multilayer resonators and metamaterials^28^,^29^,^30^, then to metamaterials with 3D resonators^31^,^32^,^33^, and, finally, to various systems and devices^10^,^16^,^34^ based on such metamaterials. Nowadays, metamaterials for THz and optical ranges are mainly fabricated by planar patterning technologies. Freedom in the designing the electromagnetic response of planar element is greatly restricted due to the absence of magnetic coupling to the in-plane magnetic field and electric coupling to normal electric field, no true chiral response of a planar element can be realized besides extrinsic chirality (i.e. when the planar element lacks an inversion centre and is tilted in such a way that the wave vector of incident radiation, the normal to the element plane and the polar vector of the element constitute a 3D-chiral triad^35^). For the most exciting applications of metamaterials, such as complete 3D invisibility cloaking, perfect lensing and others, macroscopic properties tailored in all the three dimensions are required (this problem has been discussed, e.g. in review^36^), for that 3D resonators with electromagnetic response controlled in all the three dimensions are required. Mass fabrication of such sophisticated well-tuned ordered 3D resonators with dimensions and precision needed for strong resonances in THz and optical ranges is still a very challenging task for modern technology. Several technologies were applied to make metamaterials with 3D resonators: multilayer electroplating^37^, membrane projection lithography^38^, lift-off lithography on nonplanar surfaces^39^, deep-etching^40^, shadowing vapour deposition^41^, direct laser writing^42^,^43^, glancing angle deposition^44^, three-level photolithography^45^, interferometric lithography^46^, and proton beam writing^47^ (see also reviews^31^,^33^); however, all these approaches were strongly restricted in terms of the variety and precision of possible structures or they were not suitable for mass production.

We greatly extend freedom in designing and achievable electromagnetic properties of metamaterials with a versatile set of developed approaches based on the rolling-up of 3D resonators from strained films^48^,^49^, see also reviews^50^,^51^,^52^,^53^,^54^. The main advantages of the rolling-up method are the unique precision and freedom in 3D designing, versatility of materials and scaling of 3D elements from hundreds of micrometers down to a few nanometers. We aimed to fabricate THz metamaterials, because this range is actively developed nowadays^55^,^56^,^57^,^58^ and lacks essential optical elements (for instance, wave plates) due to the absence of natural and synthetic materials with functional THz properties. The presented set of fabrication approaches allowed us to make a range of novel THz metamaterials, which cannot be made by any other presently available technology. In particular, free polymer films with embedded arrays of rolled-up resonators were formed, that substantially extends the class of flexible metamaterials^24^,^36^,^59^,^60^ and opens up the way toward the creation of metasurfaces of complex geometry and 3D metamaterials. In our study, we mainly focused on metamaterials with helical resonators. A helix demonstrate an efficient coupling both with magnetic and electric fields along the axis. Oscillations of charges along the helix axis (electric dipole) are inseparably linked with the circling oscillations (magnetic dipole), that leads to magnetoelectric polarizability. Easy tailoring of electric, magnetic and chiral responses makes a helix an object of special interest among the traditional 3D designs of metamaterial elements. Helical resonators are in demand for chiral, magnetic, negative-index^61^,^62^,^63^, “chiral nihility”^64^ and other metamaterials, which are promising for polarization transformation, imaging below the diffraction limit, invisibility cloaking, non-reflectivity and other applications. Rolled-up helices also have prospects for practical applications besides metamaterials^65^,^66^,^67^. The rolling-up method allows one to make helices with record-breaking smoothness and precision, see detailed review on the manufacturing of helical structures^68^ and reviews on rolled-up structures including helical ones^51^,^67^. Measurements in terahertz range with a Fourier-transform spectrometer and a free electron laser show a giant polarization azimuth rotation by rather sparse arrays of metal-semiconductor microhelices. A surprising finding is strong quasiperiodic peaks in the spectra of polarization of 4-fold symmetric chiral metamaterial on the semiconductor substrate. We explain these peaks by the peculiar waveguide resonance and corroborate it with experiments and semi-analytical simulations. Application of 3D printing allowed us to assemble metamaterial-based systems with further enhanced functionality. It was achieved by the interplay of different resonances (the half-wave resonance of helices, waveguide and Fabry-Perot resonances). This interplay leads to an emergence of ultrasharp quasi-periodic peaks in polarization rotation spectra: a frequency shift of THz radiation of 0.4% results in the rotation of the azimuth of transmitted radiation by 150 degrees. The 3D printing technology and the rolling-up technology perfectly complement each other and their integration makes a basis for creation of novel systems for manipulation of radiation, having prospects for mass application.

## Results

### Fabrication approaches based on the rolling-up of 3D resonators

#### Basics of the rolling-up method

**Figure 1:**
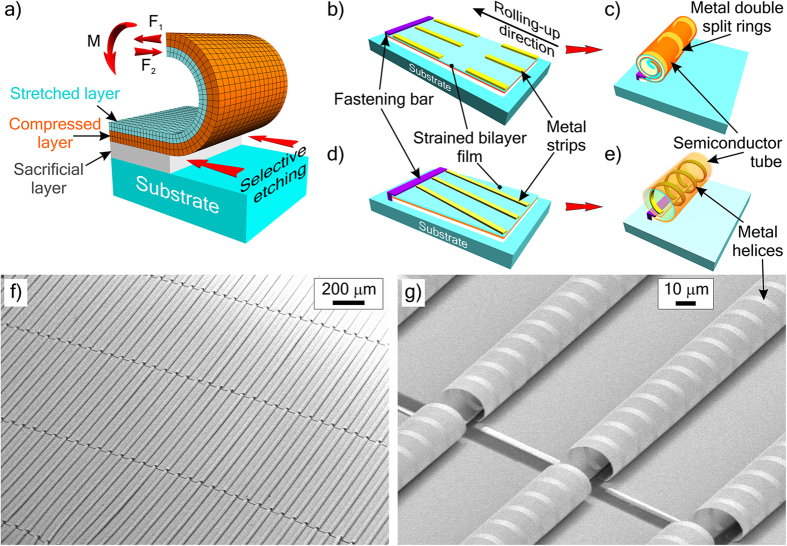
Approach 1: A tube as a carcass for metamaterial resonators. (**a**) Schematic of the strained bilayer film rolling-up into a tube: F_1_ and F_2_ are the elastic forces caused by lattice mismatch of epitaxial layers, M is the resultant bending moment of the forces. (**b–e**) Schematic formation of rolled-up carcass tubes with metal resonators: (**b**,**d**) initial planar lithographic structures, (**c**) tubes with up-right double split-ring resonators, (**e**) tubes with helical resonators (strained semiconductor or dielectric bilayer film is rolled-up altogether with the pattern of metal strips, the formed tubes are fastened to the substrate with bars of resist). (**f**,**g**) SEM–images of a chiral bianisotropic metamaterial in the form of parallel tubes (GaAs/In_0.15_Ga_0.8_As/GaAs, 5/85/20 nm) with helical resonators (Ti/Au 3.5/65 nm) on GaAs substrate.

In this work the rolling-up method for fabrication of precise 3D structures^48^, see also reviews^51^,^52^,^53^,^54^, is further developed. The basic method is as follows. Strained heterofilm elements are formed on a substrate and after detachment from the substrate they are rolled up under action of internal elastic strain between the layers (see Fig. 1а and Supplementary Video S1). The bending radius of the shell can be set from several hundred micrometers to a nanometer^48^ depending on thicknesses, strains, lithographic pattern and elastic properties of the layers. The method allows making precise 3D shells from various materials (metals, semiconductors, and dielectrics)^53^. Extreme precision is achieved for strained epitaxial pseudomorphous films. The strain in a pseudomorphous layer is proportional to the lattice mismatch, which is strictly predetermined by the composition (for instance, the lattice mismatch for InAs film on GaAs substrate is 7.2%^48^), while the epitaxial layer thickness is controlled with atomic precision. We present a diverse set of approaches adapting this rolling-up method for fabrication of metamaterials in the form of highly-ordered arrays of 3D resonant elements.

#### Rolled-up tube as a carcass for curved metamaterial elements

Figure 1b–e illustrates the formation of traditional metamaterial elements on tubular carcasses. The metal pattern is formed on the strained dielectric or semiconductor bilayer film strip (Fig. 1b,d). Being detached from the substrate this film rolls up as a tube altogether with the metal pattern (Fig. 1c,e). It should be noted that the tube diameter is predetermined by the thickness and elastic properties of the metal and by the geometry of the pattern. With the increase of the thickness or pattern density of the unstrained metal layer, the diameter of the tube increases. Here, the tube serves just as a carcass and weakly interacts with electromagnetic waves, while the curved metal elements provide a resonant electromagnetic response predetermined by their configuration. This approach allows making such elements as double split-ring resonators (Fig. 1b,c) and split-ring resonators for magnetic metamaterials, helices (Fig. 1d,e) for chiral ones and other functional elements of metamaterials. The rolling-up naturally allows forming 3D elements with precise and smooth out-of-plane magnetic loops. The main advantage of such elements over traditional in-plane lithographic elements is their effective coupling with the magnetic field of radiation in the case of normal incidence.

Figure 1f,g presents a metamaterial in the form of parallel semiconductor tubes with right-handed metal helices fabricated according to the schematics in Fig. 1d,e. Structure similar to Fig. 1d with Ti/Au metal strips, strained InGaAs/GaAs film, and AlAs sacrificial layer was prepared on GaAs substrate by epitaxy, photolithography, lift-off lithography and etching. The film elements were detached from the substrate by highly selective liquid etching of the sacrificial layer and rolled up as tubes. After liquid etching and rinsing out the etchant the structure was dried in supercritical CO_2_ to avoid collapse of tubes caused by surface tension^69^,^70^. The fabrication details are presented in the Methods Section.

To get a highly-ordered, uniform array of identical resonators, it is important to roll up all the tubes from the same side and fix them at accurately predefined mutual positions. Here it is solved with a technique using fastening bars of photoresist. Bars of photoresist block access of etchant to the sacrificial layer from the one side of the strained film strip and the strip is rolled up from the other side only. Besides that, the bars of resist keep the tubes attached to the substrate upon the completion of the rolling-up process. The gaps in tubes (Fig. 1f,g) prevent their crashing due to axial compression. To get continuous tubes a strained film with compensated strains should be used^53^, for instance In_0,64_Ga_0,36_As/In_0,43_Ga_0,57_As (50 nm/50 nm) on InP substrate.

The formed chiral structure (Fig. 1f,g) has two-fold symmetry and possesses highly bianisotropic resonant properties in terahertz range (see Measurements Section and Supplementary Information). All the helices are right-handed and parallel to each other. The mirror-image lithography pattern of metal strips results in the formation of an array of left-handed helices (see Supplementary Fig. S1). We chose one-turn helices of the so-called optimal shape, i.e. helices with pitch/radius ratio providing equality of electric, magnetic and magnetoelectric polarizabilities at operation frequency^71^. The optimal helices are very promising elements for metamaterials. They can be used to construct metamaterials with unique functional properties such as chiral metamaterial whose eigenmodes have effective refraction indices equal to ±1 and absorption for the wave with n = 1 is extremely weak^71^, non-reflecting achiral metamaterial^72^ with wave impedance matched to free space, and other metamaterials.

#### Directional rolling up of separate resonators

**Figure 2:**
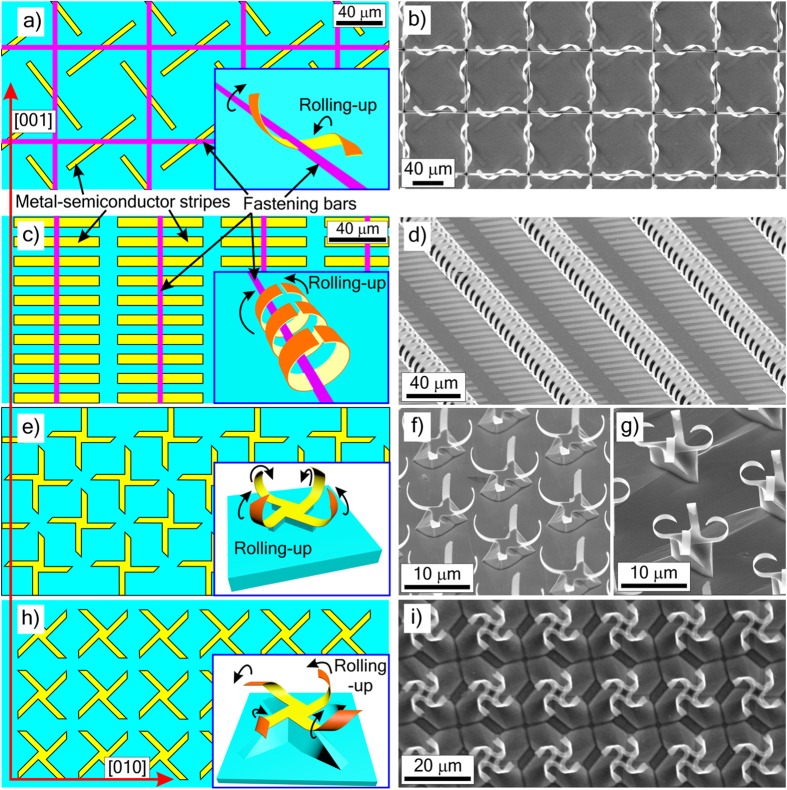
Approach 2: Directional rolling of separate elements. Schematics of lithographic arrays of strained heterofilm strips (**a**,**c**) and the transformation of the strips into helices (**a**, inset) and rings (**c**, inset) depending on the rolling direction. SEM-images of fabricated metamaterial configurations: square grid of helices (**b**) and parallel rows of up-right split rings (**d**). Helices are made of In_0.2_Ga_0.8_As/GaAs/Ti/Au (16/41/3.5/65 nm) film, rings are made of In_0.15_Ga_0.85_As/GaAs (20/95 nm) film. Schematics of the initial arrays of strained heterofilm crosses differently oriented on the substrate (**e**,**h**), transformation of a cross into a crossed split-ring element (**e**, inset) and into a propeller-like chiral element (**h**, inset). SEM-images of arrays: (**f**) crossed half-rings (In_0,6_GaAs_0,4_/In_0,4_GaAs_0,6_, 50/50 nm), (**g**) crossed split rings (In_0,47_Ga_0,53_As/In_0,64_Ga_0,36_As, 25/25 nm) on InP substrate; (**i**) chiral propeller-like elements (Si_0.65_Ge_0.35_/Si_0.85_Ge_0.15_, 16/8 nm) on Si substrate, the elements are suspended on the stems etched from the substrates. Strained film elements are yellow (top layer) and orange (bottom layer), resist is magenta, and substrate is cyan. All the initial lithographic patterns (**a**,**c**,**e**,**h**) are drawn with horizontal orientation of [010] and vertical orientation of [001] crystallographic directions (red arrows), which correspond to the energy-optimal rolling-up.

The approach using a tube as a carcass (Fig. 1) allows making out-of-plane resonators. However, in that case all the resonators are bound to an array of identical parallel cylinders that significantly limits a variety of geometries and properties. We get much more freedom in designing metamaterials with help of directional rolling-up of strained elements^52^,^53^. The directional rolling-up empowers us to transform the same 2D plane figure into 3D elements of different shapes and functionalities (Fig. 2). For instance, similar strained strips can be rolled-up as helices or as rings depending on the rolling-up direction (Fig. 2a–d). Here we used the method of directional rolling-up based on anisotropic elastic properties of strained InGaAs/GaAs films^48^,^53^. A^III^B^V^ semiconductors show a significant elastic anisotropy^73^. When a strained (100) A^III^B^V^ bilayer film is rolled up along <100> -like directions, the elastic energy is maximally released. Thus, these directions are energy-optimal for the rolling-up^48^. Initial InGaAs/GaAs film structures and the energy-optimal rolling-up directions ([010] and [001]) are shown in Fig. 2a,c,e,h. Strips of different crystallographic orientations are rolled up as helices (Fig. 2a,b), which are classical chiral elements, and as split rings (Fig. 2c,d) or closed rings (see Supplementary Fig. S2), which are magnetic elements. It is important to note that the fastening bars of resist (Fig. 2a,c) perform the following functions: keeping the helices on the substrate upon etching out of the underlying layers, positioning helices, and assisting to the desired rolling-up direction.

A square lattice of metal–semiconductor helices (Fig. 2b) is a chiral metamaterial with an isotropic in-plane response due to the 4-fold symmetry (see Measurements Section, Fig. 8), its chiral properties do not depend on polarization plane for normal incidence of radiation. The geometry of helices is chosen in accordance with the proportion suggested for the maximum rotation of the polarization plane^74^,^75^. The magnetic design of parallel up-right split rings (Fig. 2d) is highly anisotropic. Similar magnetic designs of parallel rolled-up split–rings were simulated for Au/(In)GaAs split rings in THz range^76^ and for Al split rings in IR range^77^. The latter design was also rolled-up^77^ from 2D-isotropic metal films without experimental study of its electromagnetic properties. Epitaxial pseudomorphous films with anisotropic elastic properties and fastening technique used here allow one to achieve the advanced precision, reproducibility and uniformity of arrays over the large area.

THz electromagnetic properties of parallel rolled-up split–rings made of strained (In)GaAs with metal layers were studied theoretically earlier. Presented magnetic design (Fig. 2d) can be easily implemented with a desirable gradient of ring geometries by specifying the sizes of initial strips or by partial etching of strained layers according to a desirable lithographic pattern. It is promising, for instance, for unusual beam steering simulated in^32^.

Every separate element of metamaterials presented in Figs 1f,g and 2b,d is rolled up along one crystallographic direction. We demonstrate further diversification of achievable metamaterial designs and properties with rolling-up the parts of one element in different directions. For instance, planar crosses of the same geometry but different crystallographic orientations (Fig. 2e,h) can be rolled up as magnetic crossed split rings and half-rings (Fig. 2f,g) and as chiral propeller-like elements (Fig. 2i). Here we released the strained film crosses by selective etching of the substrate and therefore the rolled-up elements (Fig. 2f,g,i) were suspended over etch pits on the stems. Anisotropic etching results in faceted etching pits. The released film is attached to the substrate along the edge of the pit. In case of an isotropic elastic bilayer film it is obvious, that the maximum release of the elastic strain energy occurs, when it is rolled up in a direction normal to the edge. Both InP (100) substrate (Fig. 2f,g) and Si (100) substrate (Fig. 2i) have pit edges oriented along [100] and [010] directions. Thus, the anisotropic etching assists the rolling–up perpendicular to edges, i.e. along <100> directions^52^. The elastic anisotropy of strained InGaAs/GaAs and SiGe/Si films also makes <100> -directions energy-optimal for the rolling-up. Thus, the desired direction of rolling-up <100> is achieved using both the anisotropic elastic properties of strained films and the anisotropic etching of substrate. For InGaAs/GaAs crosses oriented along <100> (Fig. 2e) each arm of the cross is naturally rolled up as a ring (Fig. 2e, inset). We predetermine curvature radii by the thicknesses and compositions of strained layers to get crossed split half-rings (Fig. 2f) and rings (Fig. 2g). Crossed orientation of split rings provides efficient electric and magnetic responses in the two mutually perpendicular directions, and the formed arrays have isotropic in-plane permittivity and permeability. Half-rings (Fig. 2f) have more pronounced electric and lesser magnetic response than rings (Fig. 2g). When the crosses are oriented at 45 degrees to <100> directions of the energy-optimal rolling-up (see Fig. 2h), the arms of the crosses are rolled up as helices (Fig. 2h, inset, Fig. 2i). To obtain a desired handedness for all these helices we made the ends of the crosses mitred. Mitred ends determine the initial etch pit edges and thus select the desired one of the two energy-optimal rolling-up directions. All the arms are rolled up as left-handed helices and make the 4-folded chiral element resembling a propeller (mirror-image crosses result in the formation of right-handed propellers).

#### Directional rolling up of continuously connected elements

**Figure 3:**
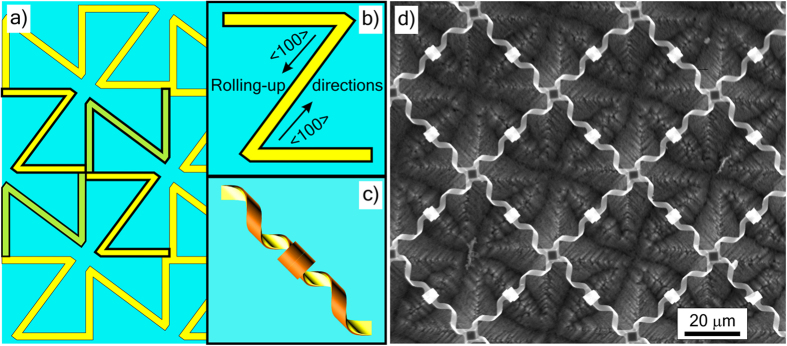
Approach 3: Directional rolling of continuously connected elements. (**a**) Schematic of the initial lithographic pattern of the strained bilayer film. The pattern can be imagined as continuously connected Z-elements (**b**), which are rolled up as two helices of one handedness connected with a ring (**c**). (**d**) SEM image of array of connected ring-helices elements (Si_0.65_Ge_0.35_/Si_0.85_Ge_0.15_,16/8 nm) on Si substrate.

The previous paragraphs were devoted to the metamaterials designed as an arrays of separate resonators fastened to a substrate. The next approach to constructing array of rolled-up elements integrates all elements into a continuous net. As an example, we present a net of continuously connected helices and rings freely-suspended on stems over the substrate (Fig. 3). The initial pattern of the strained film can be imagined as continuously connected Z-elements (Fig. 3a). Each Z –element (Fig. 3b) is rolled up as two helices connected with a ring (Fig. 3c). All the helices of the final structure are right–handed (Fig. 3d), while the rings are achiral elements. Moreover, the relative electromagnetic response of rings can be reduced to a minimum, when only the <110> -oriented strip-elements for helices are covered with additional layer of metal, while the <100> -oriented strip-elements for rings include semiconductor or dielectric layers only. A mirror-image initial pattern results in the formation of left-handed helices and an array of opposite chirality. Depending on the rolling-up direction the same Z-like pattern can be rolled as two rings connected with a helix, or as three connected helices also (see Supplementary Fig. S3).

Metamaterials with continuously connected 3D elements are promising for electrically-tunable metamaterials^34^, free-standing metamaterials^30^, and elastic metamaterials^78^ (especially with helical springs).

#### Post-rolling metallization

Resonant elements of metamaterials are usually made of metal to obtain the maximum electromagnetic response. We presented the rolled-up configurations both with metal (Figs 1f,g and 2b) and without metal layers (Figs 2d,f,g,i and 3d). The latter ones were covered with metal by chemical solution deposition of Ni and by metal-organic chemical vapour deposition (MOCVD) of Pd (see some examples in Supplementary Fig. S2) after the rolling–up. Post-rolling metallization allows one to decrease the diameters and inherits such advantages of the rolling-up of pure semiconductor epitaxial films as an extreme accuracy both of the diameter and the rolling-up direction (see details in the text accompanying Supplementary Fig. S2).

Our 3D nanofilm elements are made of undoped semiconductor materials with high refractive index (GaAs, Si), which are often used for fabrication of all-dielectric metamaterials and metasurfaces^40^,^59^. Moreover, for obtaining higher electromagnetic responses these elements can be additionally covered with dielectric similarly to post-rolling metallization.

#### Embedding of 3D elements into free-standing polymer film

For a number of practical applications metamaterials must have a flexible, transparent and thin base instead of a semiconductor substrate. For making such metamaterials, we have developed a special technology allowing one to make free-standing polymer films with embedded rolled-up 3D elements.

**Figure 4:**
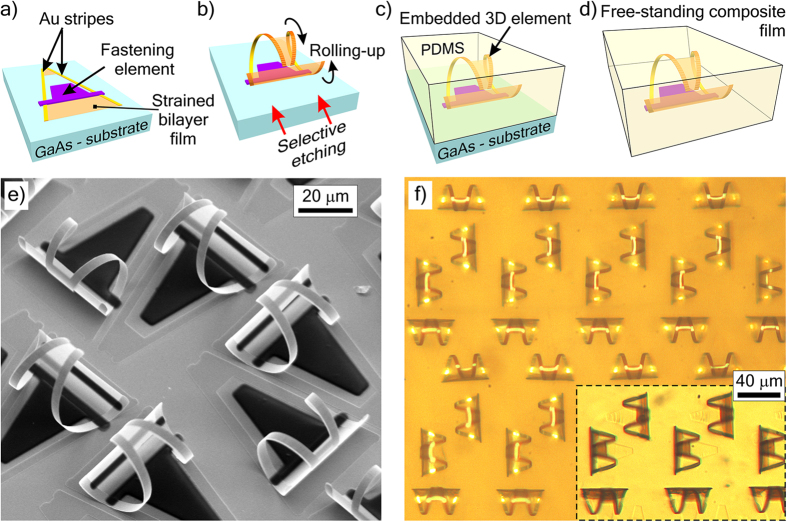
Approach 5: Embedding of rolled-up 3D elements into a polymer film. Schematic of the embedding process: (**a**) initial lithographic element, (**b**) rolled-up element fastened to the substrate with a special fastening element (violet), (**c**) 3D element in liquid silicone, (**d**) cured silicone film with embedded 3D element after detachment from the substrate. (**e**) SEM-image of the array of rolled-up elements on GaAs substrate (Ti/Au, 4/50 nm) on partially tubular carcasses (GaAs/In_0.15_Ga_0.85_As/GaAs, 6/20/75 nm). (**f**) Reflected-light and (**f**, Inset) transmitted-light photomicrographs of the same array embedded into a free PDMS film.

After the wet etching of the sacrificial layer and rolling-up of resonators (Fig. 4a,b), the sample is thoroughly rinsed in water and organic solvents without exposing it to air. Then, the liquid organic solvent is gradually replaced by a liquid pre-polymer (Fig. 4c) till complete filling of all cavities in the 3D structures. Then, the pre-polymer layer is thinned by spinning, polymerized, and detached from the substrate (Fig. 4d).

Polymers hardened by linking their short chains without solvent evaporation are preferable since in this case the volume remains unchanged and the rolled-up nanofilm elements are not deformed during polymerization. We embedded arrays of rolled-up elements into various polymer films (chemical-resistant varnish, polymethylmethacrylate (PMMA), polyimide resin and others). Polydimethylsiloxane (PDMS) was found to be the best choice for the embedding approach, because it shows very low shrinkage at polymerization, highly elastically flexible^79^,^80^, and practically transparent in THz range^81^. Due to the sufficient mechanical strength and elasticity PDMS film with thickness comparable with the diameter of the rolled-up elements is detached without any crack in contrary to all the other tested materials.

In our example after complete removal of sacrificial layer the rolled-up element (Fig. 4b,e) is fastened to the substrate only by special fastening element made of photoresist (violet, Fig. 4a–c). The trapezoidal part of the fastening element is connected directly to the substrate, while the bar-like part is connected to the free-standing rolled-up element. The fastening photoresist element is adhered to the cured PDMS much stronger than to GaAs and easily detached altogether with the hardened polymer film. To further facilitate detachment of PDMS film from GaAs substrate the sample is coated with antiadhesive monolayer of 1-octadecanethiol^82^ after the rinsing of etchant and before the replacement of rinsing solution with liquid polymer. Such composite films with area of up to 10 square centimetres were easily detached (there are no technological restrictions to make films of much larger area). Embedded 3D resonators maintain their shape, dimensions and mutual positions without noticeable deformations. Figure 4f presents micrographs of the formed free-standing PDMS film with embedded 3D resonators (InGaAs/GaAs/Ti/Au). Rolled-up nanofilm resonators are extremely elastic shells^52^,^53^,^83^, PDMS is elastically stretchable by 40–55%^80^. We elastically stretched the formed composite films about 10% of the original length multiple times, the composite films with the less rigid elements (for instance, pure helices) can be elastically stretched by even greater values.

To illustrate the embedding approach the two-dimensional weakly reflective metamaterial with compensated chirality on GaAs substrate^84^ is taken as an initial structure (Fig. 4e). This metamaterial is based on elementary resonators rolled up as pairs of optimal helices of opposite handedness (Fig. 4a,b). Due to the equal permittivity and permeability near the resonance the wave impedance is matched with air, and PDMS film with resonant metal helices (Fig. 4f) becomes almost non-reflective (the reflection does not exceed 0.02 within the range of 1.6–1.9 THz, that is close to the experimental error, while pure PDMS film reflection is >0.15 in the same frequency range).

We also made films with embedded chiral metamaterials in the form of square lattices of microhelices^85^. It should be noted that the embedding of nanofilm resonators into a polymer protects them from mechanical damage and corrosion.

In some cases (fragile polymers etc.) detachment of polymer film based on removal of additional sacrificial layer is better than simple mechanical detachment of composite film described here.

Obtained films can be transferred on the other films or substrates with desirable functional properties, for example, on tunable VO_2_ films^22^,^86^ changing electromagnetic properties at phase transition.

Thus, polymer films with embedded resonators are promising as building blocks for layer-by-layer formation of three-dimensional metamaterials and for construction of advanced functional systems.

#### Formation of metamaterial-based systems with the use of 3D printing and thermal shrinkage

We showed formation of metamaterals in the form of planar arrays of 3D rolled-up resonators, including stackable polymer composite films for fabrication of 3D arrays of resonators. This subsection is devoted to the fabrication approaches for advanced 3D arraying of 3D resonators and metamaterial-based systems with additional cavity resonators. Up-to-date additive technologies widen the freedom in spatial positioning of resonators at micrometer scale and thus enable to control the coupling between resonators. We made some simple systems with use of 3D printing.

Firstly, we attached flexible composite films to 3D printed cylindrical objects and to their PDMS mouldings by simple wrapping (3D printed objects were covered with PDMS). As a result, the flexible metamaterial strictly followed the cylindrical profile.

Secondly, we made PDMS slab with an embedded non-planar array of 3D resonators as follows. PDMS mouldings of two 3D-printed structures with matched corrugated profiles were formed. The composite film shown in Fig. 4f was placed between the two mouldings covered with liquid photo-curable PDMS with low shrinkage (see stamping process details in the Methods section). Then, pressure was applied to this stack, and the film with embedded resonators took the corrugated shape, with the liquid pre-polymer facilitating the film slipping across the humps of the corrugated surfaces. Then, PDMS was cured by exposure to UV radiation. As a result we obtained PDMS slab with embedded corrugated array of resonators. We also made corrugated array of resonators by the transfer of the flexible composite film onto a heat-shrinkable material with subsequent heating and shrinkage (see Supplementary Fig. S4).

Thirdly, to form systems with enhanced chiral properties presented in Fig. 7a and Supplementary Fig. S5 we made holder assemblies by 3D printing. The 3D printing allows making thin plane parallel air-gap between solid slabs that is crucial for such applications. Moreover, 3D printing allows flexible tailoring of the holder assembly to laboratory samples of nonstandard shapes. THz properties of these systems are presented and discussed in the next section.

In our opinion, integration of the rolling-up technology with additive technologies is a very promising direction for fabrication of functional systems with novel properties. This field opens much wider possibilities than the first attempts presented here.

### Systems with chiral metasurfaces: results of measurements

**Figure 5:**
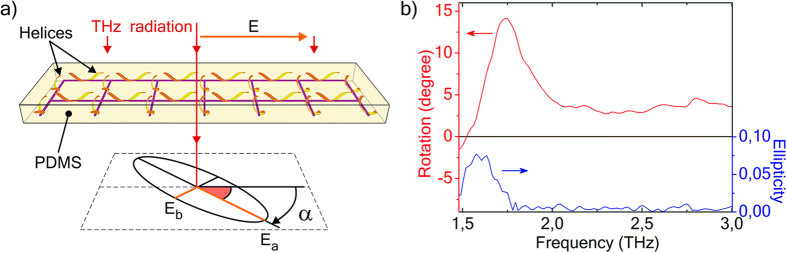
Schematic and THz spectra of the square lattice of metal-semiconductor helices embedded into a free polymer film. (**a**) Schematics of the structure and measured polarization parameters: α– angle of rotation of polarization plane, E_a_ and E_b_ are semimajor and semiminor axes of polarization ellipse. (**b**) Polarization azimuth rotation and ellipticity spectra.

We have confirmed efficient functional resonant properties of six chiral metamaterials and systems in THz range with Fourier-spectrometry and direct polarization measurements with Novosibirsk free electron laser (see details of measurements in Methods). The polarization of transmitted radiation is characterized with polarization azimuth rotation spectra and spectra of ellipticity (squared ratio of semiminor E_b_ and semimajor E_a_ axes of polarization ellipse), see Fig. 5a.

#### Square lattice of helices in polymer film

The structure schematically shown in Fig. 5a is made by embedding (See subsection *Embedding of 3D elements into free-standing polymer film)* of the square lattice of helices (Fig. 2b) into a free polymer film (PDMS). The polarization rotation and ellipticity spectra of this composite film have rather simple shapes (Fig. 5b), which are typical for a half-wave resonance. A half-wave resonance occurs when the length of an unwound helix L is about a half of the wavelength λ in the medium (2L = λ). Thus, for helices in polymer (Fig. 5a) the half-wave resonance condition is 2L = λ_polymer_ = λ_0_/n_polymer_ (λ_0_- free-space wavelength, n_polymer_- refractive index of polymer), while for helices in free space 2L = λ_0._ Due to the 4-fold symmetry of the structure its polarization rotation and ellipticity spectra (Fig. 5b) are independent from the polarization plane of normally incident radiation. A monolayer of helices rotates the polarization plane up to 14.1°, which corresponds to a giant rotatory power up to 170°/λ. As one can see in ellipticity spectrum we observe practically pure rotation of linearly polarized radiation. It is known that the chiral metamaterials for the lower frequency ranges and with the higher filling factors show greater values of both the absolute rotation of polarization plane angle and rotatory power per a wavelength (see, for instance, chiral metamaterials with bilayer resonators comprising electromagnetically coupled planar elements for GHz range^87^ and for THz range^88^.

#### Parallel helices on GaAs substrate

**Figure 6:**
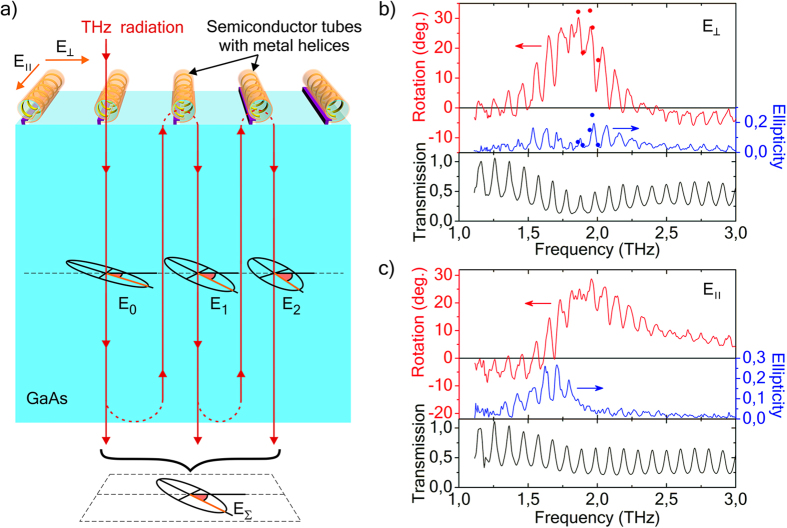
Schematic and THz spectra of the system with parallel metal-semiconductor helices (helices-GaAs). (**a**) Schematic of the system and the interference of multiple beams with different polarizations caused by the Fabry-Perot effect: the field E_0_ corresponds to the beam directly transmitted through helices, the field E_1_ – the beam having been once reflected from the helices, the field E_2_ - the beam that has been twice reflected from the helices, E_Σ_– the resultant transmitted radiation. The beams are spaced apart for better visualization. (**b**) Polarization rotation, ellipticity, and transmission spectra for incident radiation polarized perpendicular to the axes of the helices (E_⊥_), lines - Fourier-transform spectrometer data, markers - free electron laser data. (**c**) Polarization rotation, ellipticity, and transmission spectra for incident radiation polarized along the axes of the helices (E_II_).

**Figure 7:**
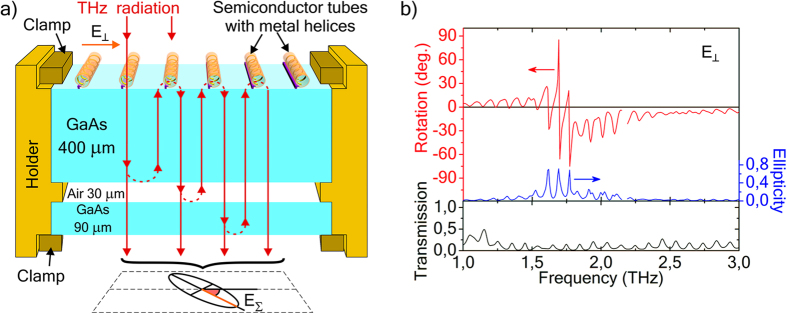
Schematic and THz spectra of the system with parallel helices and two GaAs layers (helices-GaAs-air-GaAs). (**a**) Schematics of the system and Fabry-Perot multiple beam interference, (**b**) polarization rotation, ellipticity, and transmission spectra for incident radiation polarized perpendicular to the axes of the parallel helices (E_⊥_).

**Figure 8:**
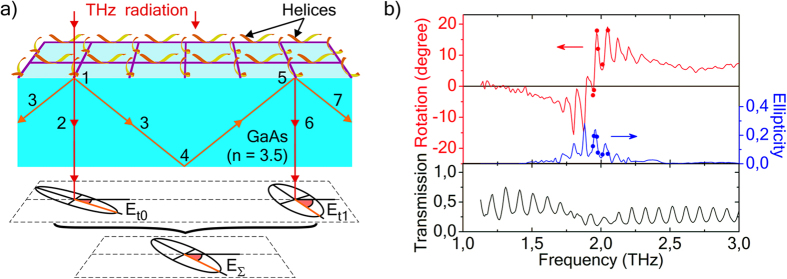
Schematic and THz spectra of the system with square lattice of metal-semiconductor helices (helices-GaAs). (**a**) Schematic of the system and the waveguide effect: 1 – excitation of helices by incident radiation, 2 – normal wave transmitted through the metasurface, 3 – the wave of the first order diffraction (the lattice of helices serves as a transmission diffraction grating), 4 – total internal reflection, 5 – repeated excitation of the helices by the guided wave, 6 – normal waves re-radiated by the helices, 7 – oblique waves re-radiated by the helices (the lattice serves as a reflection diffraction grating). E_t0_ – directly transmitted wave, E_t1_ – wave that passed one round-trip inside the substrate, E_Σ_ – result of multiple wave interference. (**b**) Polarization rotation, ellipticity, and transmission data: lines - Fourier-transform spectrometer data, markers - free electron laser data.

Systems composed of chiral metasurfaces and GaAs substrates have more complex spectra: quasiperiodic sharp peaks are superimposed on smooth half-wave resonance curves (Figs 6, 7, 8). These quasiperiodic peaks relate to the multiple reflections from the substrate boundaries and the monolayer of helices (metasurface) as it is shown below. It should be noted that GaAs is almost transparent to radiation and has rather a high refractive index in THz range (3.4–3.8).

The system based on parallel helices (see the schematic in Fig. 6a and SEM-images in Fig. 1f,g) has spectra with regular peaks (Fig. 6b,c), that is mainly caused by normal Fabry-Perot reflections (Fig. 6a). Both the polarization azimuth and ellipticity of radiation are changed at every normal reflection from helices due to their high bianisotropy. The transmitted radiation E_Σ_ is a result of interference of multiple beams with different polarizations (see Fig. 6a). The transmission, polarization rotation and ellipticity spectra of this system show the same periodicity of peaks (Fig. 6b,c). The polarization rotation achieves α~30° with maximum peak-to-peak value Δα~20°, maximum ellipticity is 0.19 for incident radiation polarized perpendicular to the axes of the helices (E_⊥_) and 0.26 for radiation polarized parallel to them (E_II_). Such a giant transformation of polarization is combined with the transmission, which is significantly high for metamaterials (peaks in transmission spectra *>0*.*5* even at resonance, Fig. 6a,b).

#### Parallel helices on double substrate (GaAs-air-GaAs)

To get more radiation coming back to helices at some frequencies and thus enhance the interaction of helices with radiation, we have introduced an additional GaAs substrate at the backside of the system with parallel helices (Fig. 7a). This enhancement of quality factor of Fabry-Perot resonator causes a dramatic change of spectra and giant sharp peaks in polarization spectra (compare Figs 7b and 6b) for incident radiation polarized perpendicular to the axes of the helices (E_⊥_): the maximum polarization rotation α = 85° with a drop to α = −65° (peak-to-peak amplitude Δα > 150°) for the frequency shift from f = 1,6924 THz to f = 1,6996 THz, i.e. for Δf/f = 0.4%. The polarization of transmitted radiation changes from almost linear one to almost circular one and back to almost linear polarization for small frequency shifts (see ellipticity spectra in Fig. 7b). Polarization rotation, ellipticity, and transmission spectra have the same period (Fig. 7b). Polarization rotation, ellipticity, and transmission spectra for incident radiation polarized parallel to the axes of helices (E_II_) are presented in Supplementary Fig. S5, their spectra show the same periodicity of peaks as well.

#### Square lattice of helices on GaAs substrate

Two 4-fold symmetric systems with identical square lattices of helices were studied: the first one embedded into a polymer film (Fig. 5a) and the second one on a GaAs substrate (Fig. 8a). They have spectra with an obvious shift of the half-wave resonance according to the shortening of the wavelength in polymer: λ = λ_0_/n_polymer_ (see Figs 5b and 8b). We were surprised to observe a quasi-periodic peak structure in the polarization rotation and ellipticity spectra (Fig. 8b) of the square lattice of helices on GaAs substrate (Fig. 8a). It cannot be explained by normal Fabry-Perot reflections, because this array of helices has 4-fold symmetry and does not change polarization at normal reflection. Moreover, the period in the transmission spectrum corresponds to normal Fabry-Perot reflections, while the periods in the polarization rotation and ellipticity spectra are equal to each other and less than the period in transmission spectrum.

The existence of these peaks in polarization spectra was corroborated by direct measurements of polarization with Novosibirsk free electron laser (see markers in Fig. 8b), the details of measurements are presented in Methods Section.

We refer this unusual phenomenon to the peculiar guided-mode resonance. Fig. 8a schematically illustrates the suggested mechanism of this phenomenon. Normally incident radiation excites the lattice of helices (1 in Fig. 8a) and they radiate into a substrate with high refractive index n~3.5. Wavelength in the substrate λ = λ_0_/n is less than the lattice period, that makes non-zero diffraction orders possible. Thus, secondary radiation of helices not only contribute to the normally transmitted waves (2 in Fig. 8a), i.e. the zeroth diffraction order, but also gives rise to oblique waves, i.e. the first diffraction order (3 in Fig. 8a). These oblique waves undergo total internal reflection at the backside of the substrate (4 in Fig. 8a) and come back to the helices (5 in Fig. 8a). The helices are excited by evanescent waves that penetrate the space above the substrate. The excited helices (5 in Fig. 8a) again radiate diffracted waves both in normal (6 in Fig. 8a) and oblique (7 in Fig. 8a) directions. The wave propagating in normal direction (6 in Fig. 8a) is partially transmitted through the whole system, while the oblique wave is further guided inside the substrate (7 in Fig. 8a). The oblique waves change their polarization at every interaction with the 4-fold symmetric lattice of helices. Therefore, the transmitted waves after different numbers of successive round trips inside the substrate (E_t0_, E_t1_, and so on) have different polarizations. Depending on the phase progression per a round trip they can interfere constructively or destructively and thus determine peaks in polarization spectra (Fig. 8b) of the resultant wave E_Σ_ (Fig. 8a).

In Fig. 8a we schematically illustrate the suggested mechanism of the process with the example of just one helix excited by incident radiation and only first order diffraction with one bouncing to the right along the row of helices, transmitted wave E_Σ_ is also shown as a result of interference of only two waves with different polarizations. The real process is more complicated. Firstly, the dipoles induced in two mutually perpendicular helices of the lattice unit cell are different, and the cells are excited simultaneously and uniformly all over the structure. Secondly, the waves diffracted on the square grating propagate in different directions, the directions out of the drawing plane are not shown. Thirdly, we have not shown the normal Fabry-Perot reflections in Fig. 8a. At normal reflections from a 4-fold symmetric structure the radiation does not change polarization, but it still significantly excites the dipoles in the helices. For this system the normal Fabry-Perot effect plays a key role in arising peaks in transmission spectra only.

Briefly, the lattice of helices acts as a diffraction grating and a waveguide coupler simultaneously. It allows the normally incident wave to couple into the guided waves and the guided waves to couple out to the normal transmitted ones. Guided waves are bouncing up and down between the metasurface side and backside of the substrate with polarization transformation at every round-trip. The waves coupled out after different numbers of round-trips have different polarization. The result of their interference strongly depends on the frequency, and it gives rise to the peaks in polarization spectra.

The principal distinction from the usual waveguide resonance^89^ is that it manifests itself in polarization spectra. In contrary to other systems with waveguide resonance our system includes an array of true chiral resonant elements. The period in polarization spectra is less than the period in the transmission spectrum. The period in polarization spectra is determined by the guided mode resonance, whereas the period in transmission spectra is determined by the normal Fabry-Perot effect. The round-trip optical path for normally reflected radiation is shorter than that for an oblique one. The shorter optical path results in the larger periods in spectra.

The mechanism proposed to explain peaks in polarization rotation and ellipticity spectra is supported by two following experiments and semi-analytical simulations. Thinning of the substrate leads to the shortening of the optical path and periods of peaks increase in accordance with the suggested mechanism (see spectra for the square lattice of helices on the thinned substrate in Supplementary Fig. S6). In the second experiment the system metamaterial-substrate was placed between two diaphragms. The second diaphragm partly cuts off the multiple waves, which are conditioning the quasiperiodic structure, and the peak-to-peak amplitude decreases (see Supplementary Fig. S7).

We simulated the polarization spectra of the square lattice of helices on the substrate (see Fig. 2b) using a semi-analytical model, and obtained quasiperiodic sharp peaks in the vicinity of the waveguide resonance (see the simulated spectra in Fig. S9 and the details of the model in Supplementary Text 1). Within the framework of the model the unit cell of the lattice consisting of two mutually perpendicular helices is taken as a point chiral element. These elements are arranged at the nodes of an infinite square lattice above a lossy dielectric slab, which simulates the substrate. Simulation results show that the periods in the polarization rotation and ellipticity spectra are equal to each other and less than the period in the transmission spectra (Supplementary Fig. S9), that agrees with measured spectra (Fig. 8b).

## Discussion and Conclusions

The main challenges in the field of metamaterials are the mass production of large-area metamaterials, the dynamic control of metamaterial properties, the fabrication of functionally more rich 3D resonators, and the scalability to micro- and nanoresonators of high precision (see a review^90^ and papers^34^,^91^). These unsolved technological problems hold back metamaterials at the stage of laboratory experiments and keep them off the practice. Against this background, consider now our results.

In the present publication, we have described a set of approaches that have allowed us to introduce a novel class of metamaterials and metasurfaces which could not be fabricated by any other presently available technology. Our approaches further develop the rolled-up technology^48^. Prominent advantages of the rolling-up strategy for making metamaterials include the possibility of mass production, parallel fabrication of resonators over large areas, diversity of realizable designs and arrangements of 3D resonators, precision and scalability of their shapes, wide selection of materials (dielectrics, semiconductors, conductors, and unique 2D materials like graphene^53^,^92^). Smooth out-of-plane magnetic loops of 3D rolled-up resonators open the way to efficient tailoring of magnetic and bianisotropic properties of metamaterials.

To illustrate the capabilities of the presented approaches, we have formed many novel THz metamaterials with diverse resonators (helices, split rings, propeller-like elements and several more sophisticated configurations) and metamaterial–based systems.

We have demonstrated the possibility to embed arrays of 3D nanofilm resonators into free polymer films, with the resonators preserving their shapes and mutual positions. The presented embedding approach substantially extends the functional capabilities of flexible metamaterials since, by now, only flexible films with simple embedded planar resonators or 3D resonators on polymer surfaces^36^ have been demonstrated. We have also demonstrated the possibility of transferring our composite films onto other substrates and objects, including curved objects being in demand for invisibility cloaks^93^,^94^, for antireflective coatings of curvilinear objects, and for other applications^36^. Layer-by-layer stacking of such films would allow one to proceed from metasurfaces to 3D metamaterials.

Measurements in terahertz range performed on a Fourier-transform spectrometer and on a free electron laser have demonstrated a giant optical activity of rather sparse arrays of helices, this property of such arrays being due to precise tuning of all resonant elements and their efficient smooth helical geometry. A number of novel systems with metasurfaces were formed and studied. A monolayer of formed microhelices rotates polarization plane by 3–5 orders of magnitude greater than natural optically active materials and by a factor of some tens greater than the best liquid crystals of comparable thickness (in wavelengths) at the frequencies of their maximum optical activity do. Moreover, in THz range both liquid crystals and natural materials show optical activities negligibly small for practical applications. Proposed metamaterials with microhelices provide a basis for the development of thin compact polarization transformation devices and elements for THz integral photonics, telecommunication, sensing and identification of biological tissues and substances, THz-imaging for medical and technical diagnostics and security (see^58^,^95^ and references in^96^,^97^). Besides passive elements such as polarization rotators, circular polarizers, absorbers, polarization converters, beam-splitters, antireflective absorbers, asymmetric transmitters we discuss ways to making active elements on metamaterials with microhelices for tunable polarization modulators, filters and switches.

The system in the form of 4-fold symmetric chiral metamaterial fabricated on a semiconductor substrate with a high refractive index has demonstrated surprising quasi-periodic peaks in polarization spectra, which were not matched with the Fabry-Perot peaks in the transmission spectra (see Fig. 8b). We have attributed the observed effect to the peculiar waveguide resonance and corroborated it with experiments and simulations.

The application of 3D printing technology has allowed the formation of more advanced systems, in which the metasurfaces based on rolled-up microresonators were integrated with slab macroresonators. The interplay of resonances in those systems leads to an emergence of extremely sharp and strong peaks in polarization spectra: one of such systems has demonstrated a maximum polarization azimuth rotation of α > 85° and a maximum peak-to-peak amplitude Δα > 150° for relative frequency shift Δf/f = 0.4%. Such sharpness of peaks can be used for continuous dynamic control of the polarization of transmitted radiation by small variations of laser frequency (see a review and a paper on frequency-tunable lasers^98^,^99^). Polarization manipulation at fixed frequency can be implemented by shifting the half-wave resonance of helices. Besides the thermal, electric, magnetic and optical effects (see a review^100^) the half-wave resonance frequency can be tuned by the small change of the refractive index of helix environment (see the shift of the half-wave resonance caused by the embedding of helices into polymer in Figs 5b and 8b). For example, a polymer composite with VO_2_ particles, that exhibit a metal-insulator phase transition, can be used as a tunable host. The resonant frequency of 3D free-standing rolled-up nanofilm elements is much more susceptible to ambient refractive index than traditional plane resonators lying flat on high-index substrates (Si, GaAs). For the latter ones the substrates with high refractive index “shunt” small alterations of the refractive index of the other half-space. Metamaterials with tunable electromagnetic properties are promising both for radiation manipulation^15^,^25^,^27^,^34^,^91^,^101^,^102^ and sensing applications^21^,^103^. The discovered peculiar chiral guided-mode effect (Fig. 8) opens up possibilities of fast and effective manipulation of radiation and creation of supersensitive systems comprising a metamaterial on a multilayer substrate with tunable layer(s) as follows. Total reflection of oblique diffracted waves plays a crucial role in the chiral guided-mode effect. The switching between the total reflection and the total transmission of oblique waves through the tunable material layer can be realized, for instance, using the resonant optical tunnelling^104^ or graphene-assisted frustrated total internal reflection^105^.

Another road to tunability is based on flexibility and stretchability of polymer films with embedded resonators. As far as our shell-resonators are made of extremely elastic nanofilms, our composite films can be greatly deformed without rupture similarly to the metamaterial films with planar split-rings^106^,^107^.

Capabilities of presented formation approaches are much wider then it is demonstrated in this paper. They can be used for fabrication of all-dielectric metamaterials (see reviews^10^,^59^,^108^), gradient metamaterials (mainly required for invisibility cloaking and other transformation optics applications), loss-free and active metamaterials^109^, metamaterials beyond electromagnetism^78^ and in other fields where highly-ordered arrays of precise 3D micro- and nanostructures are in demand (biomedicine, microfluidics, micro- and nanomechanics, nanophotonics, sensors and others). The approaches illustrated here with a range of THz metamaterials are applicable to infrared and optical metamaterials as well. All technological processes are parallel, compatible with IC technology, and can be implemented on standard equipment, which makes them appropriate both for R&D and mass fabrication of metamaterials.

## Methods

### Formation of parallel semiconductor tubes with metal helical resonators inside

Firstly, AlAs/GaAs/In_0.15_Ga_0.85_As/GaAs (40/5/20/85 nm) heterostructure was grown on a GaAs (100) substrate by molecular-beam epitaxy. Then, narrow Ti/Au strips (3.5/65 nm) were formed by lift-off lithography and metal deposition in vacuum (Ti is used for better adhesion of Au to GaAs). A pattern of wide strips, to be rolled up in tubes, was formed by photolithography and subsequent ion etching of semiconductor layers down to the substrate in BCl_3_ (the wide strips were oriented along <100>). Fastening bars of photoresist were formed by an additional photolithography. The patterned In_0.2_Ga_0.8_As/GaAs/Ti/Au heterofilm strips (Fig. 1d) were detached from the substrate by selective etching of AlAs sacrificial layer in an aqueous solution of hydrofluoric acid; under the action of the internal mechanical stress between the In_0.15_Ga_0.85_As and GaAs layers the wide strips of heterofilm rolled up as semiconductor tubes with the metal helices on the inner surface (see the schematic in Fig. 1e and the SEM-images in Fig. 1f,g). After wet etching the structure was rinsed in water and isopropanol and dried in supercritical CO_2_.

### Formation of a square lattice of metal-semiconductor helical resonators

Firstly, an AlAs/In_0.2_Ga_0.8_As/GaAs (45/16/40 nm) heterostructure was grown on a GaAs (100) substrate by molecular-beam epitaxy. Then, Ti/Au film strips (3/50 nm) were formed by lift-off lithography and metal deposition in vacuum. This pattern was transferred to all other layers of the epitaxial semiconductor film (AlAs/In_0.2_Ga_0.8_As/GaAs) by etching the sample in a standard etchant (orthophosphoric acid-hydrogen peroxide–water) down to the GaAs substrate (i.e. Ti/Au strips served as a mask). In this way, an array of AlAs/In_0.2_Ga_0.8_As/GaAs/Ti/Au strained strips was prepared on the substrate. Then, fastening bars of photoresist were formed on the sample by an additional photolithography. The prepared lithographic structure is schematically shown in Fig. 2a. In_0.2_Ga_0.8_As/GaAs/Ti/Au strips were detached from the substrate by selective etching of AlAs sacrificial layer in an aqueous solution of hydrofluoric acid; as a result, under the action of the internal stress the strips rolled up as helices (see Inset in Fig. 2a). After removal of the sacrificial layer the accurately positioned and oriented helices remained on the substrate due to the fastening bars of photoresist (see the SEM-image of the final structure in Fig. 2b). After wet etching the structure was rinsed in water and isopropanol and dried in supercritical CO_2_.

### Formation of other rolled-up structures

The other rolled-up structures (see Figs 2d,f,g,i,3d and 4e) were made in a similar way (formation of a lithographic structure with strained epitaxial film elements, rolling-up, and drying in supercritical CO_2_) with some differences in fabrication routes (thicknesses and compositions of strained layers stated in the main text, etchants, and sequence of operations).

The structures in Figs 2d,f,g,i and 3d had no metal layers, the planar patterns of strained semiconductor films (Figs 2c,e,h and 3a) were implemented by photolithography. The structures on GaAs substrates (Figs 2d and 4e) were rolled up by etching of AlAs sacrificial layer in an aqueous solution of hydrofluoric acid. The structures on InP substrates (Fig. 2f,g) were rolled up by etching of InP in 3HCl:1H_3_PO_4_ at room temperature without agitation. Before etching of InP each sample was cleaned in oxygen plasma, treated in 1HF:4H_2_O for 30 s at room temperature, and rinsed in water. The sample with a drop of water on the surface was submerged into 3HCl:1H_3_PO_4_ within 2 min after oxide removal. Pre-etching and etching procedures were similar to the ones described in^110^. The structures on Si substrates (Figs 2i and 3d) were rolled up by etching of Si in an aqueous solution of ammonium hydroxide.

### 3D printing

For 3D printing operations we used digital light processing 3D printer Carima DP110 and photopolymer 3DK-A83G.

### Stamping

For stamping the composite film with embedded array of 3D resonators a photo-curable commercially available PDMS X-34-4184 (OEM: Shin-Etsu Chemical, Japan) was used. Stamping was performed at a pressure of 20 atm applied for 20 min with nanoimprint system Eitre 6, Obducat.

### Measurements with Fourier-spectrometer

Polarization rotation, ellipticity, and transmission THz spectra (Figs 5b,6b,7b,c and 8b) were measured with Fourier-transform infrared spectrometer (Bruker IFS-66v). The sample was placed between two polarizers. The polarization azimuth rotation and ellipticity were calculated from transmittance spectra for the polarizers crossed at angles 0°, +45°,−45°, and 90°; the spectral resolution was 0.15 cm^−1^.

### Measurements with free electron laser

Direct measurements of polarization characteristics of transmitted radiation (Figs 6b and 8b) were implemented using the Novosibirsk free electron laser, the laser linewidth in the experiments was 0.2 cm^−1^. A polarizer-analyzer placed after the sample was rotated with a stepping motor drive system with simultaneous measurement of transmitted power at every step. Thus, ellipticity and rotation of polarization azimuth were obtained.

## Additional Information

**How to cite this article**: Prinz, V. Y. *et al*. Terahertz metamaterials and systems based on rolled-up 3D elements: designs, technological approaches, and properties. *Sci. Rep.* **7**, 43334; doi: 10.1038/srep43334 (2017).

**Publisher's note:** Springer Nature remains neutral with regard to jurisdictional claims in published maps and institutional affiliations.

## Supplementary Material

Supplementary Information

Supplementary Video S1
