# Supplementary material for: Terahertz metamaterials and systems based on rolled-up 3D elements: designs, technological approaches, and properties
Source: Sci Rep. 2017 Mar 3;7:43334. doi: 10.1038/srep43334 (PMC5335716; doi:10.1038/srep43334)
Supplement: Supplementary Information [file srep43334-s1.pdf]

# Supplementary Information

# to

# Terahertz metamaterials and systems based on precise rolled-up 3D elements: designs, technological approaches, properties

Victor Ya. Prinz, Elena V. Naumova, Sergey V. Golod, Vladimir A. Seleznev, Andrey A.Bocharov, and Vitaliy V. Kubarev

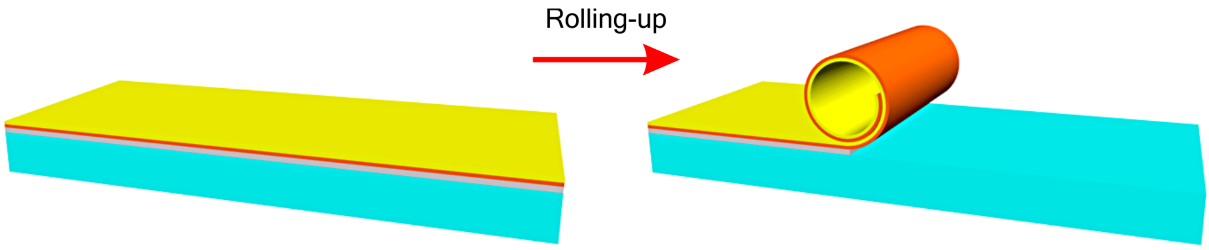


**Video S1 │ Animation of the rolling-up of a strained bilayer film**:

The initial planar structure includes a substrate (cyan), a sacrificial layer (grey), a strained film consisting of a compressed layer (orange) and a stretched layer (yellow). Removal of the sacrificial layer leads to the strained film detachment from the substrate and the film is rolled up under action of internal strains.

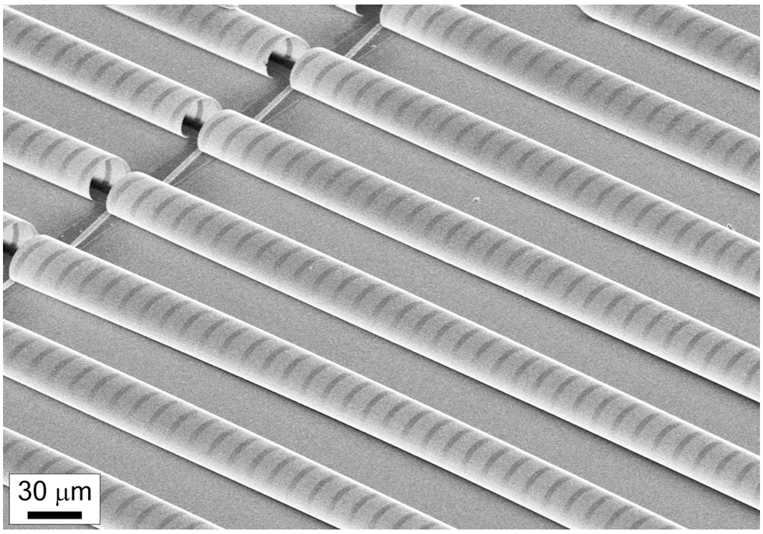


**Figure S1** │ SEM image of chiral bianisotropic metamaterial representing parallel tubes (GaAs/$In_{0.15}Ga_{0.75}As$/GaAs**,** 5/85/20 nm) with left-handed helical resonators (Ti/Au 3.5/65 nm) on GaAs substrate.

These left-handed helices and the right-handed helices presented in Fig. 1f,g are mirror-symmetric. They are rolled up from the same strained bilayer film with mirror-symmetric lithographic patterns (see schematics of the lithographic pattern and the rolling-up in Fig. 1d,e in the main text).

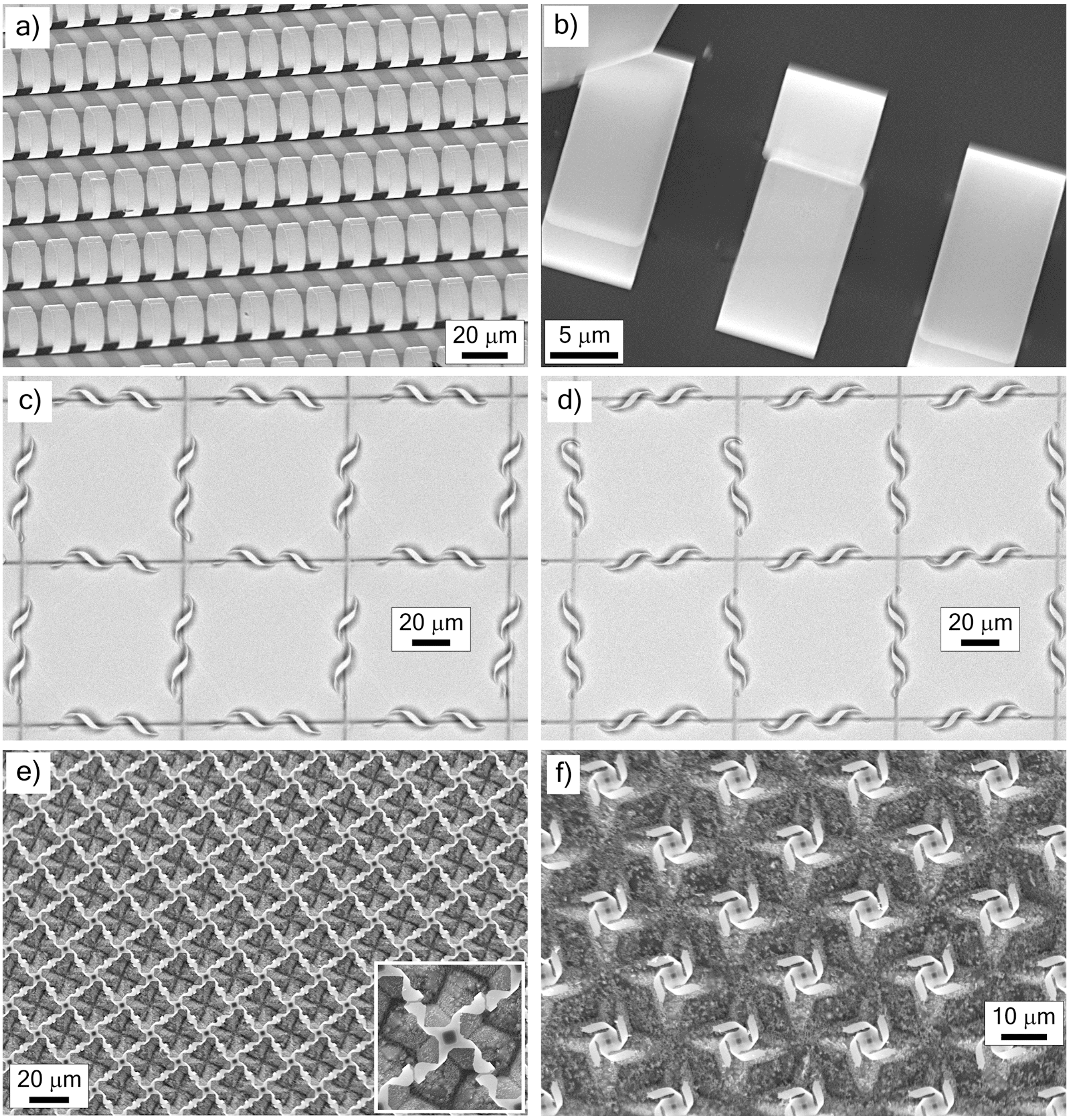


**Figure S2 │ Post-rolling metallization: SEM images of structures before and after MOCVD of Pd.** Parallel closed rings before (**a**) and after (**b**) metallization (Pd, 20 nm). Square lattices of right-handed **(c)** and left-handed **(d)** helices (Pd, 20 nm). **e,** continuous square net of rolled-up chiral elements (Pd, 18 nm), **f,** square lattice of propeller-like elements (Pd, 18 nm).

Fig. S2 presents some examples of the post-rolling metallization by MOCVD of palladium. Pd is deposited on InGaAs/GaAs elements on GaAs substrates (Fig S2 b-d) and on SiGe/Si elements on Si substrates (Fig. S2 e,f). The rolled-up elements have not been deformed during MOCVD process.

The structure in Fig. S2 a,b is similar to the structure shown in Fig 2d of the main text, but with closed rings instead of split rings. Overlapping of the ends can be controlled both by the

diameter (i.e. by thicknesses and strains of film layers) or by the lengths of the initial lithographic strips. It should be noted that the post–rolling metallization provides metal contact at the overlapping ends of the ring, while the ring rolled-up from metal–semiconductor film has a semiconductor layer in between the metal ring ends.

To make square lattices of right-handed (c) and left-handed (d) helices we used the mirror -image patterns (the pattern for right-handed helices is shown in Fig. 2a). These helices are rolled-up from the strained nanofilm similar to the one in Fig. 2b, but the film in Fig. 2b was rolled-up altogether with metal layers, while the helices in Fig. S2 c,d were covered with Pd after the rolling-up. It results in two-turn helices (Fig. S2 c,d) instead of one–turn helices (Fig. 2b) for the same length of the strip. The diameter of structure that was rolled up altogether with metal is twice more because of the metal layer elastically resists to the rolling-up of strained film. In contrary, being covered with metal after the rolling-up the helix practically does not change its diameter, as far as the metal layer does not have internal strains in this case. Post-rolling metallization approach is especially important for fabrication of metamaterials and metasurfaces for IR and optical ranges, where rolled-up resonators must have submicrometer and nanometer diameters and therefore are to be rolled up from much thinner strained films.

The square net of rolled-up chiral elements (Fig. S2, e) is rolled up from the continuous pattern of semiconductor Z-elements (see the schematics of Z- pattern and it's rolling in Fig. 3 of the main text and Supplementary Fig. S3). For structure in Fig. S2 e we used the Z-pattern with smaller period and larger ratio of the strip width to the period than for structures in Fig. 3 d and Fig. S3 g.

The square lattice of propeller-like elements (Fig. S2 f) was fanbricated using metallization of the structure similar to the one presented in Fig. 2i.

A number of methods (chemical solution deposition, atomic layer deposition (ALD), MO CVD and others) allow one to deposit metal layers on the both sides of rolled-up film elements without their deformations. The post-rolling metallization approach inherits such advantages of the rolling-up of pure semiconductor epitaxial films as the extreme accuracy both of diameter and the rolling-up direction. It should be noted that the post-rolling metallization also allows making a metal contact between the overlapping ends of 3D elements (Fig. S2 a,b).

The 3D post-rolling metallization usually covers the substrate with metal as well. For applications where the metallized substrate is of hindrance it can be removed by transfer of rolled-up elements to another substrate or by embedding them into a free transparent polymer film as described in the main text.

The used Pd precursors and MOCVD regimes were developed in the Institute of Inorganic Chemistry, Novosibirsk. SEM images in Fig. S2 are presented from permission of this scientific team.

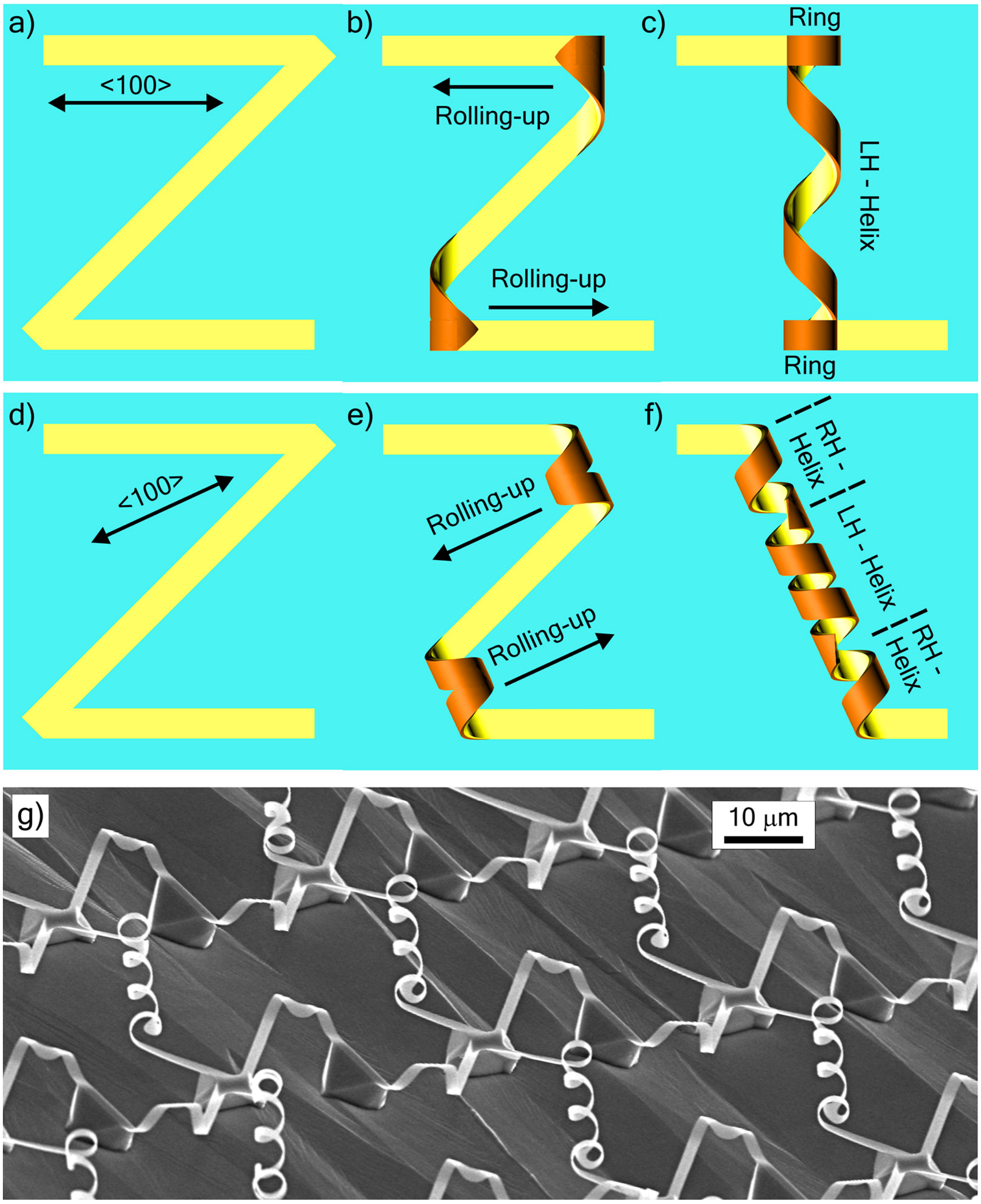


**Figure S3 | Directional rolling-up of various 3D geometries from planar Z-elements (Z-pattern is the same as in Fig. 3a in the main text).** Schematics of the directional rolling-up: **a,d** initial Z-elements, **b,e,** beginning stages of the rolling-up, **c,** resultant ring-helix-ring element, **f,** resultant element comprising three helices: right-handed helix - left-handed helix-right-handed helix, **g,** oblique view SEM-image of the structure rolled-up similar to the schematics (d-f).

Figure S3 illustrates diversity of configurations that can be rolled-up from the continuous pattern of Z-elements (Fig. 3a in the main text): ring-helix-ring (Fig. S3 a-c) and helix-helix-helix (Fig. S3 d-g, two side helices are of opposite handedness with respect to the middle one). See also Fig.3 b-d in the main text with the rolling-up of Z-elements into helix-ring-helix elements. It should be noted that the structure in Fig. S3 g is formed on InP (100) substrate. The directions [100] and [010] are not equivalent for etching of InP [P. Elias, I. Kostic, J. Soltys, & S. Hasenohrl, Wet-etch bulk micromachining of (100) InP substrates. *J. Micromech. Microeng.* **14**, 1205-1214 (2004)] in contrary to Si. This fact manifests itself in the lack of 4-fold symmetry of etching pits and therefore, of the rolled-up structures (Fig. S3, g). Here we stop the etching at the moment, when one set of parallel z-elements has been rolled–up completely and each element is freely suspended between the ends, while the perpendicular elements have not been rolled up completely and each element is additionally supported in the middle with a stem (Fig. S3 g). For comparison, see Fig. 3d in the main text with structure on Si (100): the same initial planar structure with 4-fold symmetric pattern results in the rolled-up array with 4-fold symmetry, etching pits show 4-fold symmetry as well.

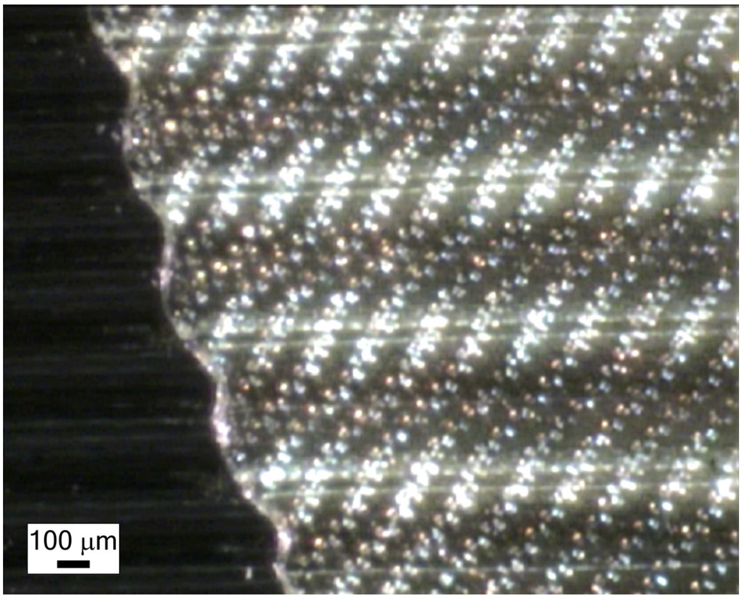


**Figure S4 │ Microphotograph of corrugated PDMS film with embedded 3D resonators on the PDMS substrate.** Period of corrugations is 400 micrometers.

We made corrugated array of resonators by the transferring the flexible composite film onto the heat-shrinkable material with subsequent heating and shrinkage. The period of corrugations is 400 micrometers.

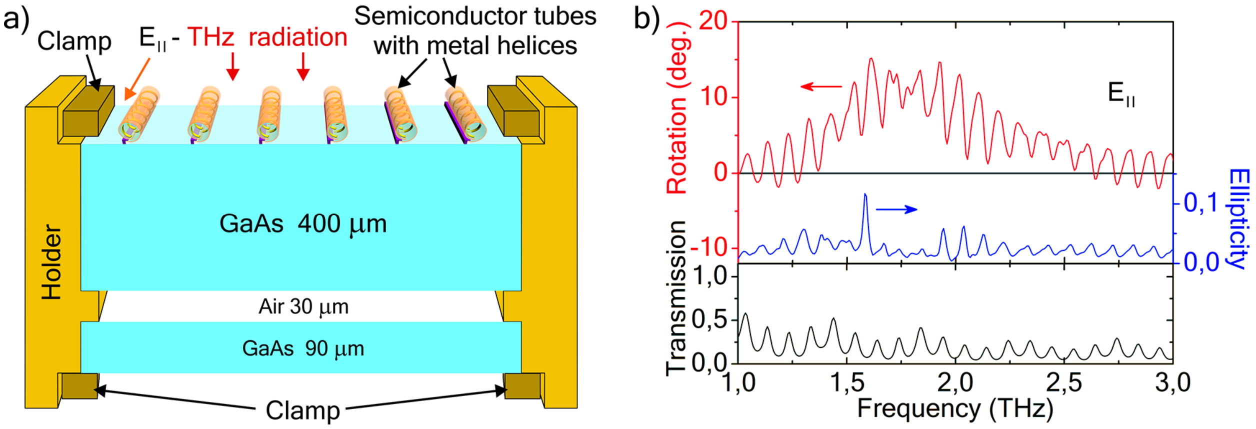


**Figure S5 │ Schematic and THz spectra of the system with parallel helices and two GaAs layers (helices-GaAs-air-GaAs). a,** schematic of the experiment and multiple beam interference, **b,** polarization rotation, ellipticity, and transmission spectra for incident radiation polarized parallel to the axes of parallel helices ($E_{II}$).

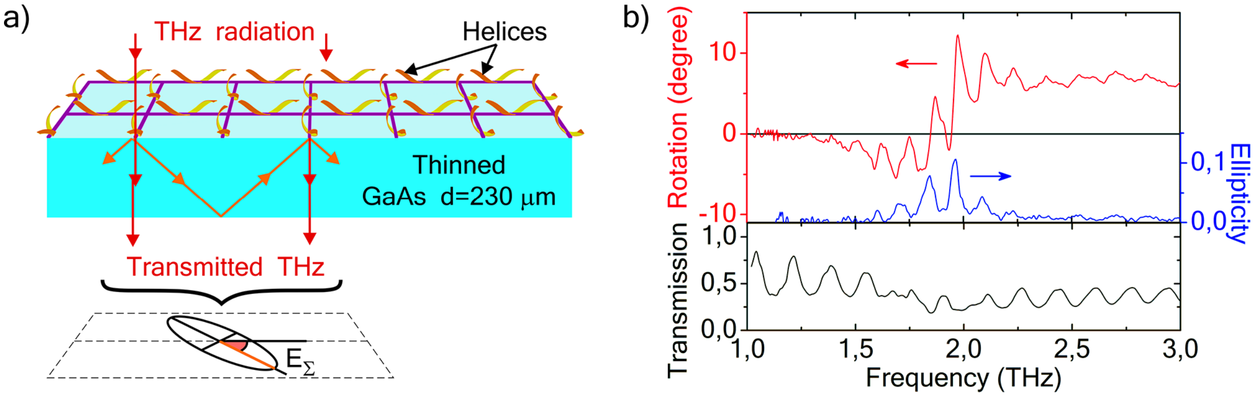


**Figure S6 │ Schematic and THz spectra of the square lattice of helices on the thin GaAs substrate (230 μm). a,** schematic of the system; **b,** polarization rotation, ellipticity, and transmission spectra.

The system (helices-substrate) in Fig. S6 a and the system in Fig. 8a of the main text differ in the thickness of the substrate only (230 μm and 440 μm, respectively). A comparison of their spectra (Fig. S6 b and Fig. 8a) show the shorter periods of quasiperiodic structure for the system with the thicker substrate in accordance with a longer wave path. Obtained results corroborate our explanation of quasiperiodic peaks in polarization spectra.

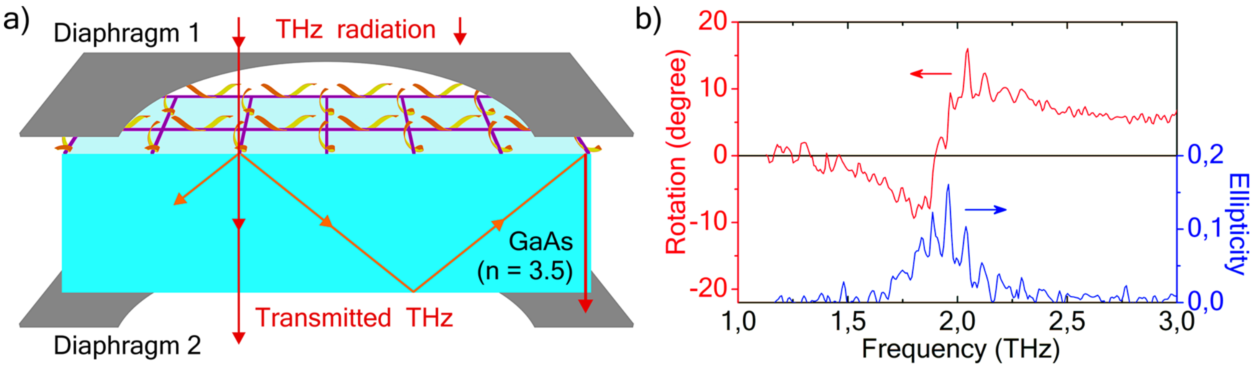


**Figure S7 │ Schematic and THz spectra of the system square lattice of helices on GaAs substrate placed between two diaphragms. a,** schematic of the experiment illustrating the cutting-off role of the second diaphragm; **b,** polarization rotation and ellipticity spectra.

Figure S7 a presents a schematic of the experiment further corroborating suggested mechanism of arising quasiperiodic peaks in the system of 4-fold symmetric lattice of helices on the substrate. When such system is placed between two diaphragms (diaphragm diameter is 1 mm) the second diaphragm restricts the number of round-trips in the substrate for the oblique waves that can be transmitted through (see schematic in Fig. S7 a). In accordance with suggested mechanism the peak-to-peak amplitude in polarization spectra decreases, and for the lower frequencies it decreases more than for higher frequencies (see Fig. S7 b in comparison with Fig. 8b in the main text).

## Supplementary Text 1

### Semi-analytical simulation of chiral metasurfaces on substrates

The scheme of the physical system under consideration is shown in Fig.S8. The polarizable elements are arranged in one plane, at the nodes of an infinite square lattice of length $l$. The above-mentioned plane is located at a distance $h_0$ from a dielectric slab of thickness $h$, permittivity $\varepsilon$ and unit magnetic permeability. A linear polarized electromagnetic wave of wavelength $\lambda$ and wavenumber $k_0 = 2\pi / \lambda$ propagating along the $z$-axis falls onto the system normally to the dielectric slab.

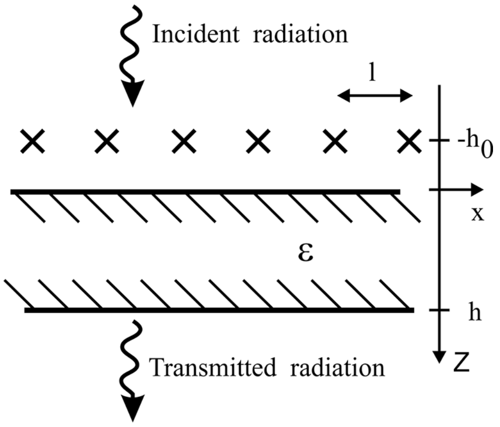


**Figure S8 │ Schematic of the physical system.** The crosses indicate the location of polarizable elements.

For wavelength $\lambda$ of the incident wave, the following inequalities hold:

$$\lambda > l, \qquad \frac{l}{\sqrt{2}} < \frac{\lambda}{n_s} < l. \tag{S0}$$

Here $n_s = \sqrt{\varepsilon}$ is the refractive index of the dielectric material. The polarizable elements are much smaller in size than $\lambda$, $h$, $h_0$, and $l$. The system is surrounded by air with air refraction index being equal to unity. The quantity $l_a$ is the absorption length of the electromagnetic wave in the dielectric layer. The problem was solved using the semi-analytical method.

All dynamic values in the problem are assumed to be complex-valued variables whose time dependence is defined by the complex-valued exponential $\exp(-i\omega t)$, where $\omega = ck_0$ and $c$ is the speed of light. In what follows, this exponential will be omitted. All equations will be written in the CGSE system.

Below, we show that all polarizable elements in the system possess identical oscillation complex-valued amplitudes of its electric and magnetic dipole moments denoted respectively as $\vec{p}$ and $\vec{m}$. The density of the electric current at the location $\vec{r}_0$ of a point polarizable element is

$$\vec{j} = \left(-i\omega\vec{p} - c\left[\vec{m}, \vec{\nabla}\right]\right)\delta(\vec{r} - \vec{r}_0)$$

where the square brackets denote the vector product.

According to [Munk, B. & Burrell, G. Plane-wave expansion for arrays of arbitrarily oriented piecewise linear elements and its application in determining the impedance of a single linear antenna in a lossy half-space. *IEEE Trans. Antennas Propag.* **27**, 331-343 (1979)], in the case of an infinite square array of synchronously oscillating currents the Green function for the vector potential is

$$G(\vec{r} - \vec{r}\,') = \frac{2\pi i}{cl^2} \sum_{\mathrm{k}} \frac{\exp\left(ik_x(x - x') + ik_y(y - y') + ik_z\,|\,z - z'\,|\right)}{k_z}$$

where the discrete set of vectors $\vec{k}$ for $\lambda > l$ is defined by the formula

$$\vec{k} = \left(\frac{2\pi n}{l}, \frac{2\pi m}{l}, k_z\right),$$
$$k_z = \begin{cases} k_0\,, \; n = m = 0 \\ i\sqrt{\left(\frac{2\pi}{l}\right)^2 \left(n^2 + m^2\right) - k_0^2}\;, \; other\ n\ and\ m \end{cases} \tag{S1}$$

with $n$ and $m$ being integer numbers. The square lattice is parallel to the plane $xy$, with the lattice lines being directed along the axes of the plane. The integration over $d^3r'$, defining the vector potential with the help of the Green function $G(\vec{r}-\vec{r}')$, is performed over one period of the lattice. From here, using the traditional formulas, the electric field vector at the point $\vec{r} = (x, y, z)$ can be calculated as the sum of the expressions for harmonic waves of the form

$$\vec{E}_k = -\frac{2\pi i}{l^2 k_z}\left( \left[\vec{k},\left[\vec{k},\vec{p}\right]\right] + k_0\left[\vec{k},\vec{m}\right] \right) \exp(ik_x x + ik_y y + ik_z\left|z + h_0\right|)\ . \tag{S2}$$

Here, we take into account the fact that the system of the polarizable elements is located in the plane $z = -h_0$. The wave vectors $\vec{k}$ are also defined by formulas (S1).

From formulas (S1) and (S2) and from the first inequality in (1) it follows that for $n = m = 0$ the system of polarizable elements emits a wave propagating along the *z*-direction. In the case of $|n| + |m| \neq 0$, the system emits evanescent waves.

Passing partially through the upper boundary of the dielectric slab, the electromagnetic waves with wave vector (S1) undergo transformation into electromagnetic waves with wave vector $n_s k_0$ and with wave vector components

$$\vec{k}' = \left(\frac{2\pi n}{l}, \frac{2\pi m}{l}, k_z'\right),$$
$$k_z' = \sqrt{\left(n_s k_0\right)^2 - \left(\frac{2\pi}{l}\right)^2 \left(n^2 + m^2\right)}. \tag{S3}$$

From inequalities (S0) and formulas (S3), one readily obtains that an evanescent wave with $|n| + |m| = 1$ emitted by the system of the polarizable elements undergoes transformation into a wave propagating in the dielectric slab. These waves propagate at the angle of total internal reflection to the faces of the dielectric slab. Evanescent waves with $|n| + |m| > 1$ transform into evanescent waves propagating in the dielectric slab. Along with conditions (1), we will consider the wavelengths $\lambda$ and slab thicknesses $h$ such that, on their return to the plane $z = 0$ after the reflection from the lower face of the slab, the evanescent waves with $|n| + |m| > 1$ strongly decay and therefore become negligibly weak in comparison of the waves propagating in the slab.

Propagating along the *z*-axis, a plane electromagnetic wave with field $\vec{E}_0 \exp(ik_0 z)$ falls onto the system formed by the polarizable elements and by the dielectric slab. In the slab, only waves with field strengths $\vec{E}_j^{\pm} \exp(i\vec{k}_j^{\pm} \cdot \vec{r})$ ($j = 1,...,5$) can be propagate. Here, the point denotes the

scalar product of vectors. The signs “+” and “-” refer to the waves that propagate toward the larger and smaller values of $z$, respectively. The wave vectors $\vec{k}_j^+$ can be determined by formulas (S3) with numbers $n$ and $m$ to be chosen according to the following rules:

$$\begin{aligned} &j=1:\ n=0,\ m=0\\ &j=2:\ n=1,\ m=0\\ &j=3:\ n=-1,\ m=0\\ &j=4:\ n=0,\ m=1\\ &j=5:\ n=0,\ m=-1 \end{aligned} \qquad \text{(S4)}$$

The vectors $\vec{k}_j^-$ differ from the vectors $\vec{k}_j^+$ just in the sign of the $z$-component. From the specified correspondence (S4) and formulas (S3), we can also determine the vectors $\vec{k}_j$.

The Maxwell equations yield the transformation matrices for the electric fields at the interface between dielectrics 1 and 2 having permittivities $\varepsilon_1$ and $\varepsilon_2$, respectively. We choose the direction of the *z*-axis along the interface-normal direction, and the direction of the *x*-axis, along the interface (as in Fig. S8). Let $\vec{E}_1 \exp(i\vec{k}_1 \cdot \vec{r})$ be the electric field strength of the wave incident from the side of dielectric 1, which propagates in the plane $xz$, $\vec{E}_2 \exp(i\vec{k}_2 \cdot \vec{r})$ be the electric field strength of the reflected wave, and $\vec{E}_3 \exp(i\vec{k}_3 \cdot \vec{r})$, the electric field strength of the transmitted wave propagating in dielectric 2. Then, we have

$$\vec{E}_2 = \hat{R}\,\vec{E}_1,\ \ \vec{E}_3 = \hat{T}\,\vec{E}_1,$$

with the transformation matrices $\hat{R}$ and $\hat{T}$ defined as

$$\hat{R} = \begin{pmatrix} -\dfrac{k_{1z}k_{1z}r_{\parallel}}{\varepsilon_1 k_0^2} & 0 & \dfrac{k_{1z}k_{1x}r_{\parallel}}{\varepsilon_1 k_0^2} \\ 0 & r_{\perp} & 0 \\ -\dfrac{k_{1x}k_{1z}r_{\parallel}}{\varepsilon_1 k_0^2} & 0 & \dfrac{k_{1x}k_{1x}r_{\parallel}}{\varepsilon_1 k_0^2} \end{pmatrix},\quad \hat{T} = \begin{pmatrix} \dfrac{k_{3z}k_{1z}t_{\parallel}}{\varepsilon_2 k_0^2} & 0 & -\dfrac{k_{3z}k_{1x}t_{\parallel}}{\varepsilon_2 k_0^2} \\ 0 & t_{\perp} & 0 \\ -\dfrac{k_{3x}k_{1z}t_{\parallel}}{\varepsilon_2 k_0^2} & 0 & \dfrac{k_{3x}k_{1x}t_{\parallel}}{\varepsilon_2 k_0^2} \end{pmatrix} \qquad \text{(S5)}$$

where

$$r_{\perp} = \frac{k_{1z}-k_{3z}}{k_{1z}+k_{3z}},\ \ t_{\perp} = \frac{2k_{1z}}{k_{1z}+k_{3z}},\ \ r_{\parallel} = \frac{k_{1z}/\varepsilon_1 - k_{3z}/\varepsilon_2}{k_{1z}/\varepsilon_1 + k_{3z}/\varepsilon_2},\ \ t_{\parallel} = \frac{2k_{1z}/\varepsilon_1}{k_{1z}/\varepsilon_1 + k_{3z}/\varepsilon_2} \qquad \text{(S6)}$$

and

$$k_{3x} = k_{1x},\ k_{3z} = \pm\sqrt{\varepsilon_2 k_0^2 - k_{3x}^2},\ \ k_{1z} = \pm\sqrt{\varepsilon_1 k_0^2 - k_{1x}^2}$$

being the transmission and reflection coefficients for TE and TM waves, respectively. The above expressions are also valid for the complex-valued wave vectors. The sign of the roots is to be chosen according to the direction of propagation of the waves along the *z*-axis. For imaginary values of $k_{1z}$ and $k_{3z}$ the sign of the root should comply with the condition of decay of the waves propagating away from the interface between the dielectrics.

In the case of wave vectors not lying in the plane $xz$, for the transformation matrices of electric field vectors, instead of expression (S6) transformed matrices $\hat{O}^T\hat{R}\hat{O}$ and $\hat{O}^T\hat{T}\hat{O}$ are to be

used, where $\hat{O}$ is the matrix of rotation around the $z$-axis through the angle of deflection of the vector $(k_{1x}, k_{1y}, 0)$ from the $x$-axis. In this case, in formulas (S5) and (S6) $\sqrt{k_{ix}{}^2 + k_{iy}{}^2}$ must be used in place of $k_{ix}$. Next, we denote the matrices $\hat{O}^T\hat{R}\hat{O}$ and $\hat{O}^T\hat{T}\hat{O}$ for waves with wave vectors $\vec{k}_j^{\pm}$ ($j = 1,...,5$) falling onto the interface from the side of the dielectric respectively as $\hat{R}_j^{\pm}$ and $T_j^{\pm}$ ($j = 1,...,5$). For the waves falling onto the interface from the side of the air with wave vectors $\vec{k}$ defined by formula (S3) the matrices $\hat{R}'_{nm}$ and $\hat{T}'_{nm}$ can be determined in a similar way. Next, we denote the matrices $\hat{T}'_{nm}$ with subscripts $n$ and $m$ defined by expression (S4) as $\hat{T}'_j$ ($j = 1,...,5$).

Under the adopted assumptions, we formulate the condition of wave reflection from the lower internal boundary of the dielectric slab as

$$\vec{E}_j^- = \exp\left(2(ik_{jz}^+ - \gamma_j)h\right)\hat{R}_j^+\vec{E}_j^+, \quad j = 1,...,5 \tag{S7}$$

The exponential factor is caused by the position of the lower boundary with the coordinate $z = h$. To allow for the weak decay of the waves in the dielectric, we introduce the damping constants $\gamma_j$:

$$\gamma_1 = \frac{1}{2l_a}, \; \gamma_2 = \gamma_3 = \gamma_4 = \gamma_5 = \frac{1}{2l_a\cos(\theta)},$$

where $l_a$ is the absorption length of the waves in the dielectric layer and $\theta$ is the angle of inclination of the waves to the $z$-axis.

At the upper internal boundary of the plate, we have:

$$\vec{E}_j^+ = \hat{R}_j^-\vec{E}_j^- + \vec{S}_j + \begin{cases} t_0\vec{E}_0, & j = 1 \\ 0, & j > 1 \end{cases}, \quad j = 1,...,5. \tag{S8}$$

Here, the first term is caused by the reflection of the waves at the upper boundary of the plate. The second term in expression (S8) determines the contribution due to the waves emitted by the system of the polarizable elements. The third term is the contribution due to the wave falling onto the system. In formula (S8), $t_0 = 2/(1+n_s)$ is the transmission factor for the wave falling normally onto the dielectric layer.

To express vectors $\vec{S}_j$, the amplitudes of the electric field due to polarizable elements (S2) at the factor $\exp(i\vec{k}\vec{r})$ can be represented in matrix form as $\hat{C}_{nm}\vec{p} + \hat{D}_{nm}\vec{m}$. For the subscripts $n$ and $m$ complying with the subscript $j$ under rules (S4), the above matrices will be denoted as $\hat{C}_j$ and $\hat{D}_j$. Then, we have:

$$\vec{S}_j = \hat{T}'_j\hat{C}_j\vec{p} + \hat{T}'_j\hat{D}_j\vec{m}, \quad j = 1,...,5 \tag{S9}$$

For closing equations (S7)-(S9), a relationship between the fields applied at the locations of polarizable elements and the induced electrical $\vec{p}$ and magnetic $\vec{m}$ dipole moments is required. The spatial coordinates of the polarizable elements are given by the vector

$\vec{r} = (l\,n, l\,m, -h_0)$ with integer numbers $n$ and $m$. All the waves falling onto the system of polarizable elements have wave vectors of form $\vec{k} = (2\pi n_1 / l, 2\pi m_1 / l, k_z)$ with integer numbers $n_1$ and $m_1$; that is why the phase factor of the electromagnetic field at the locations of the chiral elements has the form

$$\exp(i\vec{k} \cdot \vec{r}) = \exp(2\pi\, inn_1 + 2\pi\, imm_1 - ih_0 k_z) = \exp(-ih_0 k_z),$$

which does not depend on the location of the chiral elements. Hence, the electric and magnetic dipole moments of all the polarizable elements can be represented as

$$\begin{aligned} \vec{p} &= \hat{\alpha}_{ee}\vec{E}_\Sigma - i\hat{\alpha}_{em}\vec{H}_\Sigma \\ \vec{m} &= i\hat{\alpha}_{me}\vec{E}_\Sigma + \hat{\alpha}_{mm}\vec{H}_\Sigma \end{aligned} \tag{S10}$$

where $\vec{E}_\Sigma$ and $\vec{H}_\Sigma$ are the total strengths of the electric and magnetic fields, respectively, at the location of the polarizable elements. For the tensors in (S10), the relation $\hat{\alpha}_{em}^T = \hat{\alpha}_{me}$ holds.

The total electric field at the points of interest can be represented as

$$\vec{E}_\Sigma = \sum_{j=1}^{5} \exp(ik_{jz}h_0)\hat{T}_j\vec{E}_j^- + \vec{E}_s + \vec{E}_r + \left(\exp(-ik_0h_0) + r_0\exp(ik_0h_0)\right)\vec{E}_0 \,. \tag{S11}$$

Here, the expression with the summation symbol stands for the contribution due to the fields of the waves in the dielectric slab which leave the plate through its upper boundary; $\vec{E}_s$ is the strength of the electric field due to the polarizable elements that surround the selected polarizable element; and $\vec{E}_r$ is the strength of the electric field that arises as a result of the reflection of the polarizable-element-emitted waves from the upper face of the slab. The last term in (S11) is the contribution due to the wave falling onto the system together with the magnitude of the field due to the same wave reflected from the upper face of the slab; in this term, $r_0 = -(n_s - 1)/(n_s + 1)$ is the reflection factor for the wave falling normally onto the slab from the side of the air.

With the above designations, we write

$$\vec{E}_r = \sum_{n,m} \exp(2ik_z(n,m)h_0)\hat{R}'_{nm}\left(\hat{C}_{nm}\vec{p} + \hat{D}_{nm}\vec{m}\right). \tag{S12}$$

Here, for the components of $k_z$ formula (S1) is to be used.

In calculating the field $\vec{E}_s$, we use the formula for the electric field emitted by one polarizable element

$$\vec{E} = \frac{k_0^2\exp(ik_0r)}{r}\left(\left(1 + \frac{i}{k_0r} - \frac{1}{k_0^2r^2}\right)\vec{p} - \left(1 + \frac{3i}{k_0r} - \frac{3}{k_0^2r^2}\right)\vec{n}(\vec{n},\vec{p}) - \left(1 + \frac{i}{k_0r}\right)\left[\vec{n},\vec{m}\right]\right). \tag{S13}$$

In (S13), $r$ is the distance from the radiating element to the point of observation and $\vec{n}$ is the unit vector directed from the radiating element toward the observation point. Using the designation $r_{nm} = l\sqrt{n^2 + m^2}$ and taking into account the symmetry in the location of polarizable elements, through summation of the vectors determined by formula (S13) we obtain the

following expressions for the electric and magnetic fields at the location of the polarizable elements:

$$\vec{E}_s = \hat{\Pi}\,\vec{p},\ \vec{H}_s = \hat{\Pi}\,\vec{m}\,, \qquad \text{(S14)}$$

where

$$\hat{\Pi} = 4k_0^2 \sum_{\substack{n\geq 0\\ m\geq 1}} \frac{\exp(ik_0 r_{nm})}{r_{nm}} \left( \left(\hat{I} - \hat{A}\right) + \left( \frac{i}{k_0 r_{nm}} - \frac{1}{k_0^2 r_{nm}^2} \right)\left(\hat{I} - 3\hat{A}\right) \right),$$

$\hat{I}$ is the unit matrix and $\hat{A} = diag(0.5, 0.5, 0)$.

Using the Maxwell equations for the plane harmonic waves with the wave vector $\vec{k}$ defined as $\left[\vec{k}, \vec{E}\right] = k_0 \vec{H}$, one can deduce from the obtaining equation an analogous expression for the field $\vec{H}_\Sigma$ entering the previous equation.

Formulas (S7)-(S12), (S14) and the expression for $\vec{H}_\Sigma$ (not explicitly written here) form a closed system of linear algebraic equations for the unknown quantities $\vec{p}$, $\vec{m}$, $\vec{E}_j^-$, and $\vec{E}_j^+$ ($j = 1,...,5$); this system was solved numerically using standard algorithms.

To simulate polarization spectra of a square lattice of helices (see Fig 2b) we consider a unit cell of the lattice consisting of two perpendicular helices as a point chiral element. The polarizability tensor of this cell in the model with two principal elements was treated similarly to [I.V. Semchenko *et al.* Study of the properties of artificial anisotropic structures with high chirality. *Crystallogr. Rep.* **56,** 366-373 (2011)]. Neglecting all other elements in the tensors, we have

$$\hat{\alpha}_{ee} = \begin{pmatrix} \alpha & 0 & 0 \\ 0 & \alpha & 0 \\ 0 & 0 & 0 \end{pmatrix},\ \hat{\alpha}_{em} = \hat{\alpha}_{me} = \begin{pmatrix} \alpha\beta & 0 & 0 \\ 0 & \alpha\beta & 0 \\ 0 & 0 & 0 \end{pmatrix},\ \hat{\alpha}_{mm} = \begin{pmatrix} \alpha\beta^2 & 0 & 0 \\ 0 & \alpha\beta^2 & 0 \\ 0 & 0 & 0 \end{pmatrix}, \qquad (2)$$

and

$$\beta = \frac{\omega r^2 q}{2c}\,,$$

where $r$ is the helix radius, $|q|=2\pi H$, $H$ is the pitch, and $c$ is the speed of light. Here, $\hat{\alpha}_{ee}$ and $\hat{\alpha}_{em}$ are the dielectric and magnetic polarizability tensors, and $\hat{\alpha}_{em}$ and $\hat{\alpha}_{me}$ are the pseudo tensors describing the chiral properties of the helices. The chiral electromagnetic properties of the helix originate from its geometry: the translational motion of electrons along the helix axis is inseparably linked with the circling along the turns of the helix. Thus, an electric field directed along the helix axis induces not only an electric dipole moment but, also, a magnetic dipole moment also directed along the helix axis so that, here, there arises a magnetoelectric polarizability. The two diagonal elements of the pseudotensor of magnetoelectric polarizability (2), $\hat{\alpha}_{me\,xx}$ and $\hat{\alpha}_{me\,yy}$, describe the helices oriented along x and along y.

The frequency dependence of the polarizabilities is adopted in accordance with the model of a damped linear harmonic oscillator:

$\alpha \sim 1/(\omega_0^2 - \omega^2 - I\,\Gamma\,\omega)$,

where Γ is the doubled damping ratio, $\omega_0$ is the half-wave resonance frequency of the helix, $\omega_0 \approx 2\pi * c/\lambda_0$, and $\lambda_0 = 2 * l_0$, where $l_0$ is the length of the unwound helix.

The results of the semi-analytical modelling of a square lattice of helices on GaAs substrate are presented in Fig. S9. It is important to note that the periods in the ORD and CD spectra are identical, and they differ from the period in the transmission spectra.

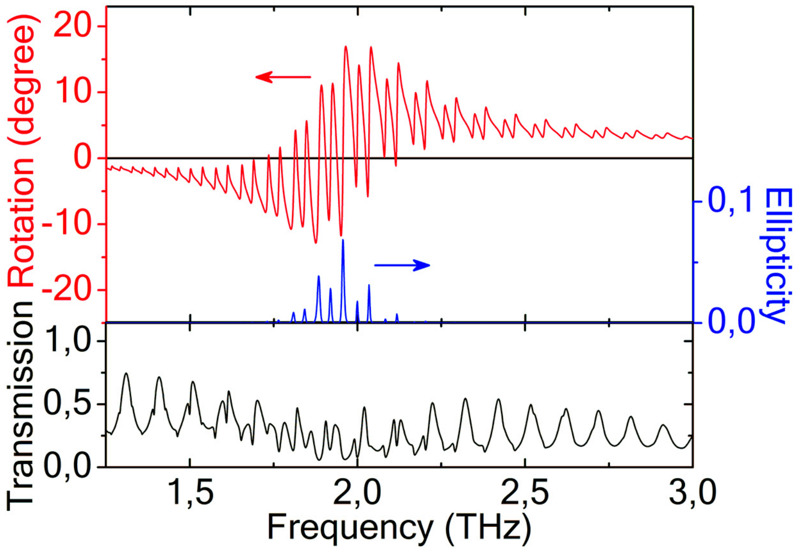


**Figure S9 │ Polarization rotation, ellipticity, and transmission spectra of a square lattice of helices on GaAs substrate calculated within the framework of the semi-analytical model.**
